# Evidence that keratinocyte microvesicle particles carrying platelet-activating factor mediate the widespread multiorgan damage associated with intoxicated thermal burn injury

**DOI:** 10.1093/jleuko/qiae078

**Published:** 2024-03-26

**Authors:** Rushabh P Lohade, Chad Brewer, Christine M Rapp, Karen M Henkels, Wenfeng Zhang, Anita Thyagarajan, Shikshita Singh, Pranali Manjrekar, Taskin Sabit, Ravi P Sahu, Jeffrey B Travers

**Affiliations:** Department of Pharmacology and Toxicology, Wright State University, 3640 Colonel Glenn Highway, Dayton, Ohio 45435, United States; Department of Pharmacology and Toxicology, Wright State University, 3640 Colonel Glenn Highway, Dayton, Ohio 45435, United States; Department of Pharmacology and Toxicology, Wright State University, 3640 Colonel Glenn Highway, Dayton, Ohio 45435, United States; Department of Pharmacology and Toxicology, Wright State University, 3640 Colonel Glenn Highway, Dayton, Ohio 45435, United States; Department of Pharmacology and Toxicology, Wright State University, 3640 Colonel Glenn Highway, Dayton, Ohio 45435, United States; Department of Pharmacology and Toxicology, Wright State University, 3640 Colonel Glenn Highway, Dayton, Ohio 45435, United States; Department of Pharmacology and Toxicology, Wright State University, 3640 Colonel Glenn Highway, Dayton, Ohio 45435, United States; Department of Pharmacology and Toxicology, Wright State University, 3640 Colonel Glenn Highway, Dayton, Ohio 45435, United States; Department of Pharmacology and Toxicology, Wright State University, 3640 Colonel Glenn Highway, Dayton, Ohio 45435, United States; Department of Pharmacology and Toxicology, Wright State University, 3640 Colonel Glenn Highway, Dayton, Ohio 45435, United States; Department of Pharmacology and Toxicology, Wright State University, 3640 Colonel Glenn Highway, Dayton, Ohio 45435, United States; Department of Dermatology, Wright State University, 125 University Blvd., Dayton, Ohio 45435, United States; Department of Medicine, Dayton VA Medical Center, 4100 W Third St, Dayton, Ohio 45428, United States

**Keywords:** acid sphingomyelinase, ethanol intoxication, microvesicle particles, thermal burn

## Abstract

Thermal burn injuries can result in significant morbidity and mortality. The combination of ethanol intoxication with thermal burn injury results in increased morbidity through an exaggerated inflammatory response involving many organs. Recent studies have linked involvement of the lipid mediator platelet-activating factor (PAF) in the pathology associated with intoxicated thermal burn injury (ITBI). The present studies tested the roles of PAF and the elevated levels of subcellular microvesicle particles (MVP) generated in response to ITBI in the subsequent multiorgan toxicity. First, thermal burn injury of HaCaT keratinocytes preincubated with ethanol resulted in augmented MVP release, which was blocked by inhibiting the PAF-generating enzyme cytosolic phospholipase A_2_ and the PAF receptor (PAFR). Second, ITBI of mice resulted in increased proinflammatory cytokine production and neutrophilic inflammation in multiple organs, which were not present in mice deficient in PAFRs or the MVP-generating enzyme acid sphingomyelinase (aSMase). Moreover, the increased bacterial translocation from the gut to mesenteric lymph nodes previously reported in murine ITBI was also dependent on PAFR and aSMase. MVP released from ITBI-treated keratinocytes contained high levels of PAFR agonistic activity. Finally, use of topical aSMase inhibitor imipramine following ITBI attenuated the widespread organ inflammatory response of ITBI, suggesting a potential therapeutic for this condition. These studies provide evidence for PAF-enriched MVP generated in skin, which then act on the gut PAFR, resulting in bacterial translocation as the mechanism for the multiorgan dysfunction associated with ITBI. Inasmuch as aSMase inhibitors are widely available, these studies could result in effective treatments for ITBI.

## Teaser

The current studies suggest the exaggerated systemic inflammatory response from intoxicated thermal burn injury involves microvesicles released from the skin carrying the lipid mediator platelet-activating factor.

## 1. Introduction

Thermal burn injuries are an important source of morbidity and mortality, with approximately 100,000 patients hospitalized in the United States annually due to severe burn injuries.^1,2^ Not only does thermal burn injury result in local effects, systemic consequences have been described, indicating that skin keratinocytes can generate bioactive agents that can travel systemically in response to damage.^3–7^ Of interest, the combination of ethanol intoxication and thermal burn injury not only is commonly encountered, but also results in increased morbidity and even mortality in comparison with thermal burn injury alone.^1,8,9^ Intoxicated thermal burn injury (ITBI) has been modeled in mice, and it appears to accurately depict human pathology.^10–15^ Of note, ethanol given to mice via oral gavage or via intraperitoneal injection prior to burn injury results in similar effects.^16^ Within 24 h of ITBI, mice develop increased inflammation, as evinced by the elevated expression of proinflammatory cytokines and leukocyte infiltration in the lung, as well as in other organs, including liver and kidneys.^10,12,15^ Murine studies have implicated the cytokine IL-6, and bacterial translocation from the intestines in response to myosin light chain kinase (MLCK) activation in the systemic multiorgan inflammatory responses from ITBI.^14,17^ Yet, the exact mechanism(s) by which ITBI involving skin keratinocytes triggers a gut response are not clear.

Defined as irregularly shaped submicron (∼100 to 1,000 nm) subcellular vesicles released from cellular membranes via exocytosis, microvesicle particles (MVP) (or large extracellular vesicles) have drawn considerable interest as potential signaling effectors. Generation of MVP occurs in response to numerous stimuli, most involving cell damage and intracellular calcium mobilization.^18–20^ In the keratinocyte and other cell types, translocation of the enzyme acid sphingomyelinase (aSMase) has been demonstrated to mediate MVP release in response to many disparate agents from G protein–coupled receptor activation to phorbol esters to ionizing radiation to ultraviolet B (UVB) radiation.^21–25^ Recent studies from our group using in vitro epithelial cell and in vivo murine models have shown that the combination of ethanol and thermal burn injury results in increased MVP release.^26^

Following release into the circulation (blood and tissue), MVP can then exert functional effects dependent on the stimuli and cell of origin. It is presumed that MVP target cells via surface membrane interactions resulting in the fusion of the MVP with the target cell membrane, thus allowing delivery of the intravesicular contents. The functions of MVP in physiological and pathological processes are believed to be dependent on their carried contents, which include bioactive lipids, nucleic acids, and proteins.^18,19,27^ One outcome of this process is that metabolically unstable compounds that are travelling inside MVP can be somewhat protected from enzymatic degradation.

The most potent lipid mediator yet described, Platelet-activating factor (PAF) was first named by Benveniste et al.^28^ in the 1970s as a lipid-soluble substance released by activated leukocytes, which was a potent platelet aggregator. The structure of this PAF was determined in 1979 and found to reside in *sn-1* ether–linked glycerophosphocholine (GPC) containing a short-chain fatty acid (e.g., acetate) at the *sn-2* position.^29^ The enzymatic synthesis of PAF is via 2 separate pathways.^30–32^ The remodeling pathway is associated with cellular stimulation from multiple sources that often involve intracellular calcium mobilization. This pathway consists of phospholipase A_2_ (often cytosolic PLA_2_) generating a lyso-GPC, which is then acetylated by an acetyl-CoA–dependent acetyltransferase (LPCAT) to form PAF. PAF can also be generated by a de novo pathway.^30^ Finally, PAF and multiple other closely related lipids can be generated nonenzymatically by free radical attack of unsaturated *sn-2* fatty acids found on a GPC.^33^ Once generated, PAF is quickly metabolized by PAF acetyl hydrolases (PAF-AHs) that cleave the *sn-2* acetyl moiety resulting in the biologically inert lyso-GPC.^34^ The half-life of PAF is short (few minutes), due to both serum- and cell-associated PAF-AH.^34,35^ PAF exerts its effects via a single G protein–coupled receptor (the PAFR), which is expressed on multiple cell types to include almost all leukocytes and epithelial cells.^32^ The PAF family of lipid mediators has been found to be involved in many processes from acute inflammatory responses to delayed immunosuppression.^30,32,36^ Moreover, PAF has been demonstrated to be generated in response to many environmental stressors including thermal injury.^22,36,37^

Recently, our group has demonstrated that activation of the keratinocyte PAFR results in MVP release.^21–24^ Of note, UVB radiation, which also is a potent generator of PAF agonists via both enzymatic and nonenzymatic processes,^33,38^ induces MVP in a PAFR-dependent manner.^21,23^ Thermal and cold injuries also trigger enzymatic PAF synthesis.^37^ Ethanol treatment of keratinocytes alone does not result in significant PAF agonistic formation, yet it augments the enzymatic production of PAF induced by UVB radiation or thermal burn injury.^26,39,40^ Consistent with the combination of ethanol + thermal burn injury resulting in increased PAF production, we have also reported using an epithelial cell line KB with/without PAFR (KBM/KBP) and wild-type (WT) and also PAFR-deficient (*Ptafr−/−*) mice, the combination of which also increased MVP release in a PAFR-dependent manner.^22,26^ The goal of the present studies is to further characterize the roles of the PAFR and MVP-dependent enzyme aSMase in the systemic multiple organ dysfunction associated with murine ITBI. Finally, we test the ability of an inhibitor of this process to assess if this intervention can thwart the systemic pathologies known to occur following the common combination of ethanol and thermal burn injury.

## 2. Methods

All chemicals were obtained from Sigma-Aldrich unless indicated otherwise. The human keratinocyte–derived cell line HaCaT and nasopharyngeal-derived KB cells were grown in Dulbecco's modified Eagle's medium high-glucose media with 10% FCIII, 6 mM L-glutamine, and a 100 μg/mL mixture of penicillin and streptomycin, as described.^22,23^ PAFR-negative KB cells were rendered PAFR-positive (KBP) by transducing with the MSCV2.1 retrovirus encoding the human leukocyte PAFR and the PAFR-deficient (KBM) by transducing with the MSCV2.1 vector alone as described previously.^41^ All cell lines were used between passages 70 and 100 and were regularly tested for mycoplasma. KBP and KBM cells were grown to approximately 50% confluence with small colonies and HaCaT cells were grown to approximately 80% to 90% confluence in 10-cm dishes. Cells were washed 3 times with Hanks’ Balanced Salt Solution (HBSS) and then incubated with prewarmed (37 °C) HBSS with 10 mg/mL fatty acid–free bovine serum albumin (BSA). Thermal burn injury was performed by placement of the cell culture dish onto a 90 °C water bath for various times.^22,37^ For studies involving ethanol, cells were incubated with 1% v/v ethanol in HBSS + BSA for 30 min before other stimulation as per our previously published studies that optimized ethanol effects on HaCaT and KB cells.^26,39,40^ For studies involving the cPLA_2_ inhibitor pyrrophenone, a concentration of 1 μM was used.^42^ This concentration of pyrrophenone did not induce a toxic response in cells as measured by trypan blue dye exclusion tests at 24 h (not shown). Imipramine was used at a concentration of 100 μM as previously reported.^22–24^

### 2.1 Use of small interfering RNA to knock down PAFR expression in HaCaT cells

HaCaT keratinocytes were exposed to 1 μL of scramble or PAFR small interfering RNA (Qiagen) along with 3.75 μL Lipofectamine (Invitrogen) and incubated for 24 h before use. Success of the knockdown was tested by quantitative real-time polymerase chain reaction comparing PAFR messenger RNA (mRNA) vs GADPH levels.

### 2.2 Mice

Female C57BL/6 WT mice (PAFR expressing; age 6 to 8 wk) were purchased from Envigo. PAFR knockout (KO) (*Ptafr−/−*) mice on a C57BL/6 background, generated as previously described,^43^ were a kind gift of Professor Takao Shimizu (Department of Biochemistry, University of Tokyo). aSMase-deficient (*Smpd1*+/−) heterozygous mice originally from Dr. Edward Schuchman's laboratory^44^ were obtained from Dr. Irina Petrache's group at the National Jewish Medical Center in Denver, Colorado. The aSMase KO (*Smpd1−*/−) mice were bred by heterozygous littermates. All mice were used at approximately 7 to 10 wk of age for the experiments. All mice were housed under specific pathogen-free conditions and kept on a 12-h light/dark cycle with free access to standard animal chow and water in the animal facility at the Wright State University. All procedures were approved by the Institutional Animal Care and Use Committee of Wright State University.

### 2.3 Thermal burn injury in murine skin

Thermal burn injury was performed using our previously published methodology.^22,26,39,40^ Wild-type or *Ptafr−*/− or *Smpd1−/−* C57BL/6 mice were anesthetized with ketamine/xylazine (100 and 10 mg/kg, respectively) and fur removed from dorsal back skin. Mice were administered 2.4 g/kg of 20% ethanol intraperitoneally. After 30 min, the dorsal skin of the mice was treated with 8-s exposure of 2 1 × 1 cm stainless steel metal blocks heated to 90 °C, resulting in ∼12% to 15% body surface area burns. Following the injury (including sham), the mice were given buprenorphine subcutaneously and were given 0.5 mL of warmed normal saline intraperitoneally immediately afterward. At 4 h posttreatment, the mice were euthanized and exsanguinated. Skin biopsies of the burned area were collected for MVP isolation. Blood was collected from the heart into lithium heparin tubes and allowed to clot for 30 min at room temperature, followed by centrifugation at 2,000 *g* for 10 min at 4 °C. Blood plasma was carefully transferred into a new tube and immediately processed for MVP isolation.

### 2.4 Tissue harvesting

The mice were sacrificed by CO_2_ narcosis on day 1 (24 h) after injury. The lungs, distal part of the small intestine, liver, kidney, and spleen were aseptically removed and one-third was placed in 10% buffered formalin for 1 h and then stored in 70% ethanol and sent to AML Laboratories for processing for routine histology. Samples were then embedded in paraffin, sliced into sections at 5 μm, and treated with hematoxylin and eosin (H&E) staining. Images of the stained sections were captured using BioTek Cytation 5 and an Olympus BX51 Microscope (in conjunction with an Olympus DP73 digital camera using CellSens software) at various magnifications (40×, 200×, 600×). Furthermore, neutrophils were counted in blinded fashion at a magnification of 600× using a confocal microscope. Three microscopic fields were examined on each slide, and each group consisted of 6 to 8 mice. The identification of neutrophils was based on their distinctive morphological characteristics, which included a multilobed nucleus and small, pale-stained granular cytoplasm. One-third of the tissues were stored in RNAlater (Thermo Fisher Scientific) overnight at 4 °C, the next day it was transferred to −80 °C for further analysis. RNA was isolated from tissue samples stored in RNAlater using a Gene JET RNA purification kit from Thermo Fisher Scientific (catalog number: 01306122). The concentration of RNA was determined using NanoDrop (Thermo Fisher Scientific), and RNA with an A260/280 ratio was used to generate complementary DNA through reverse transcription using a kit from Bio-Rad. Quantitative real-time polymerase chain reaction was performed to measure the levels of inflammatory cytokines, and gene-specific primers were used in duplicate for each response. The results were then analyzed using the 2−ΔΔCt method with actin as the endogenous control, and the expression was calculated by comparing the fold change relative to the sham-treated group. See Supplementary Table 1 for the list of primers used. The final one-third of the tissues were placed at −80 °C for future studies.

### 2.5 Isolation and measurement of MVP

MVP were collected from culture medium as previously described with slight modifications.^22–24^ In brief, cell culture medium and mice blood plasma were collected and centrifuged at 2,000 *g* for 20 min to remove cells and debris. Supernatant then transferred to a new tube centrifuge at 20,000 *g* for 70 min. The resulting pellet contained the isolated MVP. For skin biopsies, tissue was cut up finely in the microcentrifuge tube and digested in 0.5 mL of 5 mg/mL collagenase and dispase solution made in 1:1 deionized water and filtered phosphate-buffered saline (PBS), shaken overnight at 37 °C. After overnight digestion, samples were centrifuged at 2,000 *g* for 20 min to remove tissues, then followed with 20,000 *g* centrifugation for 10 min to remove remaining tissue and subcellular component. MVP from the sample supernatants were then pelleted at 20,000 *g* by centrifugation for 70 min. The concentration of the MVP was determined by using a NanoSight NS300 instrument (Malvern Instruments) exactly as previously reported.^21–25^ Three 30-s videos of each sample were recorded and analyzed with NTA software version 3.0 (Malvern Instruments) to determine the concentration and size of measured particles with corresponding standard error.

### 2.6 Bacterial translocation analysis

One day after the injury, 3 to 5 mesenteric lymph nodes were removed from each mouse, placed in cold PBS, and kept on ice. The nodes were isolated from the surrounding connective tissue and then homogenized using a frosted slide in 1 mL PBS. Homogenates (200 μL) were plated on tryptic soy blood agar plates in triplicate and incubated overnight at 37 °C. The Epson Perfection V550 Photo was used to capture images of the plate. The bacterial colonies that grew on agar plates were counted, averaged, and divided by the total number of mesenteric lymph nodes harvested. The plates were considered negative for the presence of bacteria if they did not exhibit any bacterial growth for up to 48 h.

### 2.7 Measurement of PAFR agonistic activity

The presence of PAFR agonists in lipid extracts derived from HaCaT cells was assessed by enzyme-linked immunosorbent assay as the ability of lipid extracts to induce IL-8 release in PAFR-expressing KBP cells but not in PAFR-deficient KBM cells, as described.^22,23^ Lipid extracts were isolated either from burn-treated HaCaT cells, total cell medium at various time points (using water, methanol, and methylene chloride [1:1:1 v/v] exactly as reported previously),^22,23,33^ and HaCaT-induced MVP or MVP-depleted supernatant at 5 and 120 min. Lipids were added to PAFR overexpressed KBP cells. The ratio of IL-8 released by treated KBP cells compared with 1 nM CPAF positive control–treated KBP cells were used to determine the PAFR agonistic activity level.

### 2.8 Statistics

All statistical calculations were performed using GraphPad Prism 6 (GraphPad Software). All experiments were repeated at least 3 times in separate experiments. Statistical significance was determined using 1- or 2-way analysis of variance and the post hoc Holm-Sidak method, with alpha = 5%.

## 3. Results

### 3.1 Thermal burn injury results in increased MVP release in HaCaT keratinocytes

**Fig. 1.**
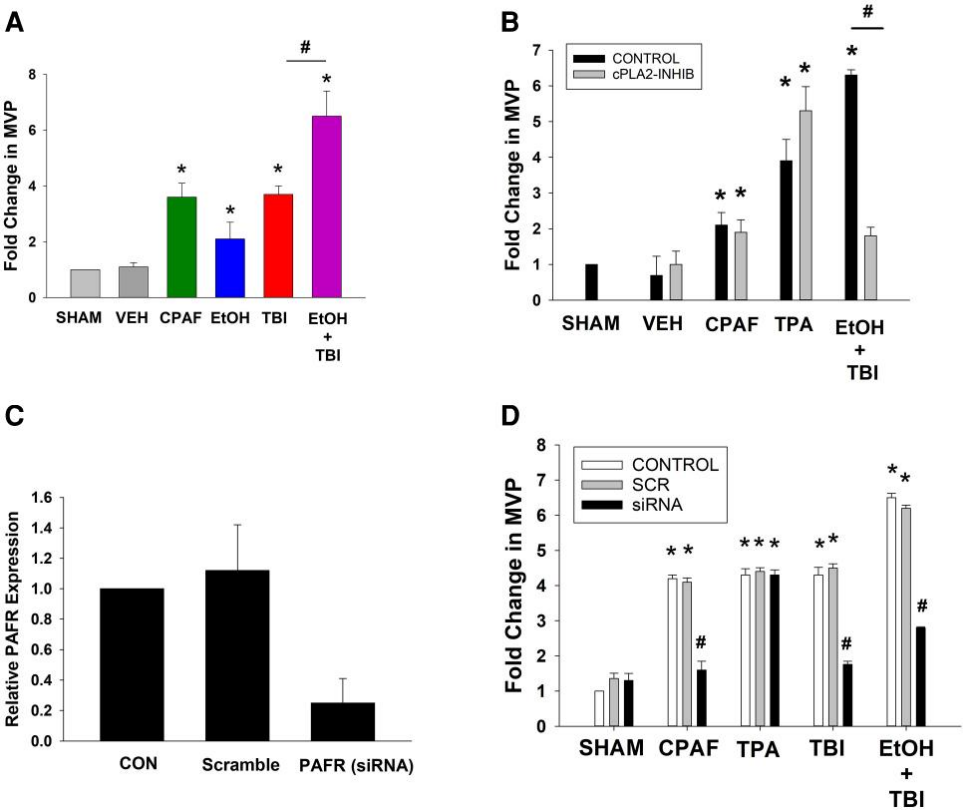
ITBI generates MVP in HaCaT keratinocytes and role of the PAF system. (A) MVP release studies. HaCaT cells were subjected to 90 °C water bath × 30 s (thermal burn injury [TBI]), 1% ethanol alone or given 30 min before TBI, or treatment with 100 nM CPAF, or 0.1% ethanol vehicle control. At 4 h, the supernatants were removed, and MVP were isolated by differential centrifugation and measured (see Methods). (B) Effect of cPLA_2_ inhibitor on MVP release. HaCaT cells were pretreated with sham control or 1 μM of the cPLA_2_ inhibitor pyrrophenone for 30 min. The cells were then exposed to 100 nM CPAF or 100 nM TPA or 1% ethanol followed 30 min later by TBI as in panel A. At 4 h, the supernatants were removed and MVP measured. (C) HaCaT keratinocytes were treated with PAFR small interfering RNA (siRNA) or scrambled, and 48 h later PAFR mRNA levels were measured by quantitative real-time polymerase chain reaction using actin to normalize levels. (D) HaCaT cells from panel C underwent treatment with various agents and MVP measured in supernatants after 4 h. The data are mean ± SD MVP fold change from at least 3 separate experiments with duplicate samples. Statistical significance was assessed using 2-way analysis of variance. Statistically significant (*P < 0.05, **P < 0.01, and ***P < 0.001) changes in MVP levels from control values. Statistically significant (^#^*P* < 0.01) differences between similarly treated samples.

We have previously reported results of in vitro and in vivo studies demonstrating that thermal burn injury stimulates enzymatic PAF production, which is enhanced in the presence of ethanol.^37,40^ Inasmuch as PAFR activation is a potent stimulus for MVP generation, our first studies assessed the role of the PAF system in the combination of ethanol and thermal burn injury–induced MVP release. For these studies, we utilized the human keratinocyte–derived cell line HaCaT.^45^ The current experiments took advantage of our previously published studies that provided concentration- and time-responsive relationships between UVB radiation and CPAF on MVP release in HaCaT keratinocytes to use 4 h as the optimal time studied.^22–24,39,40^ Shown in Fig. 1A, increased MVP release was seen in HaCaT keratinocytes in response to the PAFR agonist CPAF, ethanol, and burn injury. The combination of ethanol and burn injury resulted in increased MVP in comparison with either stimulus alone. To assess the role of the PAF system in augmented MVP release from the ethanol + thermal burn injury response, we pursued 2 strategies. First, given that ethanol + thermal burn injury results in increased PLA_2_ activity,^40^ we tested the effect of the cPLA_2_ inhibitor pyrrophenone on MVP release. Second, we used a small interfering RNA approach to diminish PAFR expression levels in HaCaT keratinocytes. As shown in Fig. 1B, pretreatment of HaCaT keratinocytes with an effective concentration of pyrrophenone^42^ inhibited the MVP release in response to ethanol + thermal burn injury yet did not affect MVP generation in response to PAFR agonist CPAF or the PAFR-independent phorbol ester stimulus. Similarly, knockdown of the PAFR by ∼70% was achieved in HaCaT keratinocytes (Fig. 1C), which diminished MVP release in response to thermal burn injury alone (Fig. 1D), as we have previously reported using KB cells with/without the PAFR (KBP, KBM).^22,40^ Knockdown of the PAFR in HaCaT keratinocytes also blocked the MVP release in response to ethanol + thermal burn injury, and CPAF had no effect on that triggered by the phorbol ester TPA (Fig. 1D). These studies confirm the role of the PAF system in ITBI-mediated increased MVP generation in a keratinocyte cell line.

### 3.2 ITBI results in increased lung inflammation in mice

**Fig. 2.**
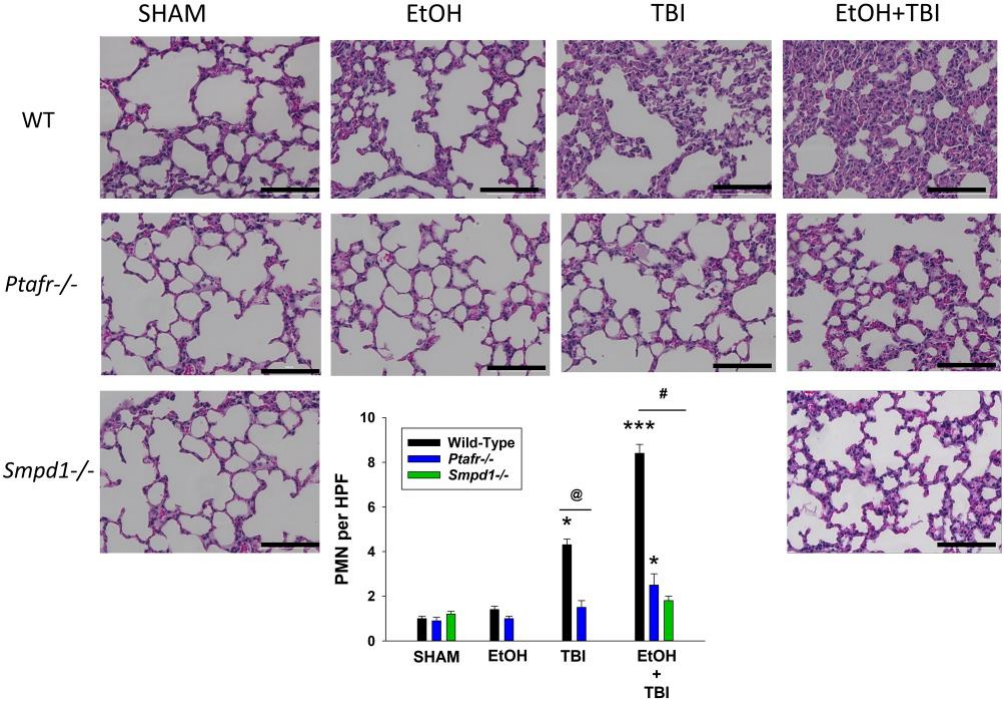
Decreased pulmonary inflammation in PAFR KO and aSMase KO mice following ITBI. One day after the injury (24 h), lungs from mice were harvested and prepared for H&E staining. The lung sections were examined for the degree of inflammation following ITBI in WT, PAFR KO (*Ptafr−/−*), and aSMase KO (*Smpd1−/−*) mice. Images were captured at magnification of 200× from 6 to 8 mice per group (scale bar = 100 μm). (Inset) PMN counts from 3 high-power fields from 6 to 8 mice taken at 600× magnitude. Statistical significance was assessed using 2-way analysis of variance. Statistically significant (*P < 0.05, ***P < 0.001) changes in PMN levels from control values (^@^*P* < 0.05, ^#^*P* < 0.01) and differences between similarly treated WT and KO mice are denoted symbolically.

Previously, we reported that the combination of ethanol and thermal burn injury resulted in increased MVP in the skin and blood of mice within 4 h,^26,40^ and 14 h posttreatment, increased levels of the cytokine interleukin (IL)-6 were measured.^40^ In these murine studies, the use of PAFR-deficient mice revealed that the MVP release and the IL-6 levels were PAFR dependent.^40^ We next demonstrate in Supplementary Fig. 1 that the increased MVP in skin following ITBI noted in WT mice^26^ was not seen in aSMase-deficient mice. Given that 1 d post-ITBI mice have been reported to exhibit increased inflammation in multiple organs,^12–17^ the next studies examined the roles of the PAFR and the key MVP-generating enzyme aSMase in this process. As shown in histologic pictures found in Fig. 2 (200×; high power) and Supplementary Figs. 2 (40×; low power) and 3 (600×; oil immersion), we found increased acute inflammatory cells in the lungs of WT mice that underwent ITBI 24 h previously. This inflammatory response was blocked in mice lacking PAFR or aSMase. The neutrophil has been described as a key effector for ITBI-induced organ responses.^13,14^ Neutrophils were quantified in the histologic sections at 600× (see Supplementary Fig. 3) and mRNA levels of myeloperoxidase were measured in lung tissue, which revealed increased numbers of these cells. The increased polymorphonuclear leukocyte (PMN) response from ITBI was attenuated in PAFR- and aSMase-deficient hosts (Fig. 2).

**Fig. 3.**
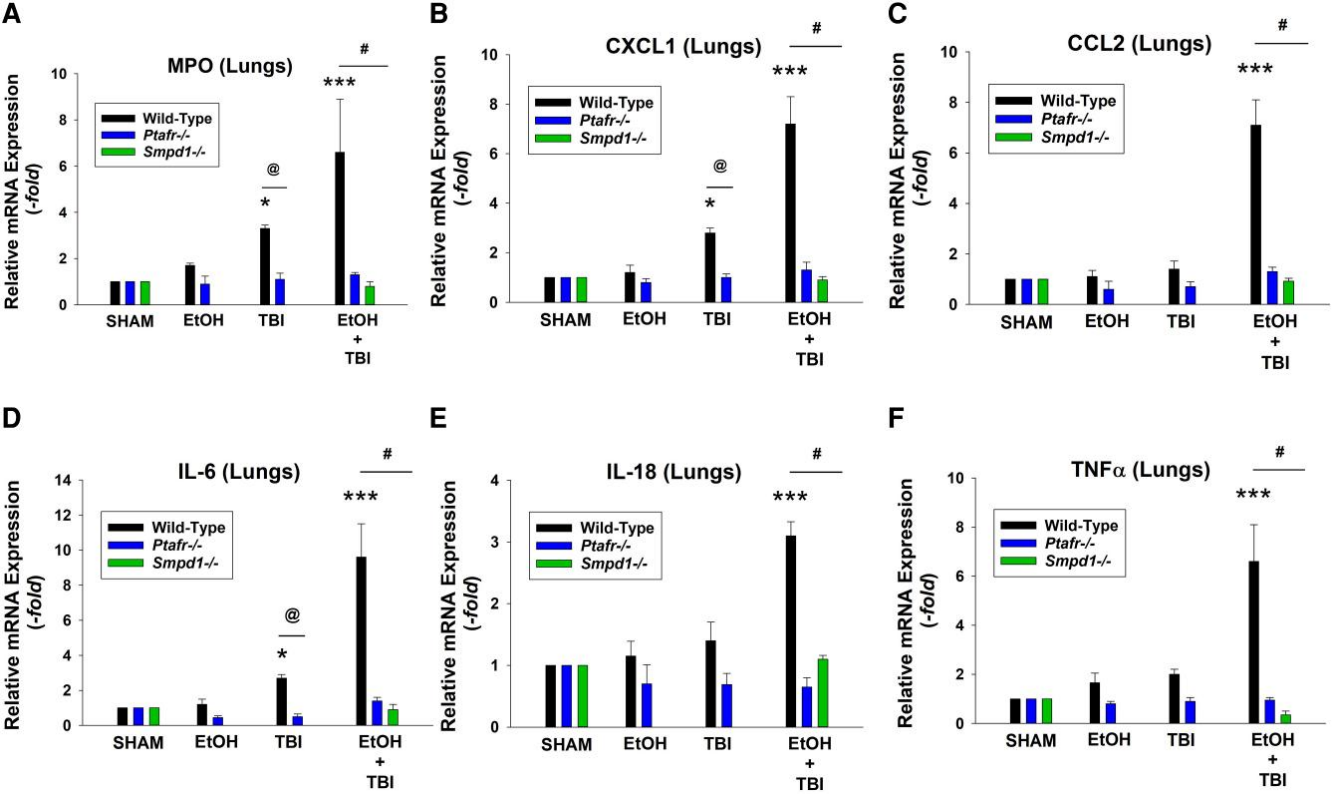
Decreased pulmonary myeloperoxidase (MPO) and cytokine expression levels in PAFR KO and aSMase KO mice following ITBI. Mice underwent treatments as in Fig. 2, and expression levels of mRNA of (A) MPO and (B–F) representative cytokines in the lungs were determined by quantitative real-time polymerase chain reaction, and the mean values with SEM were calculated from 10 to 15 mice in each group. The statistical analysis was performed using 2-way analysis of variance, with statistical significance denoted as *P < 0.05 and ***P < 0.001 compared with control values, with ^@^*P* < 0.05 and ^#^*P* < 0.01 indicating significant differences between similarly treated WT and KO mice.

**Fig. 4.**
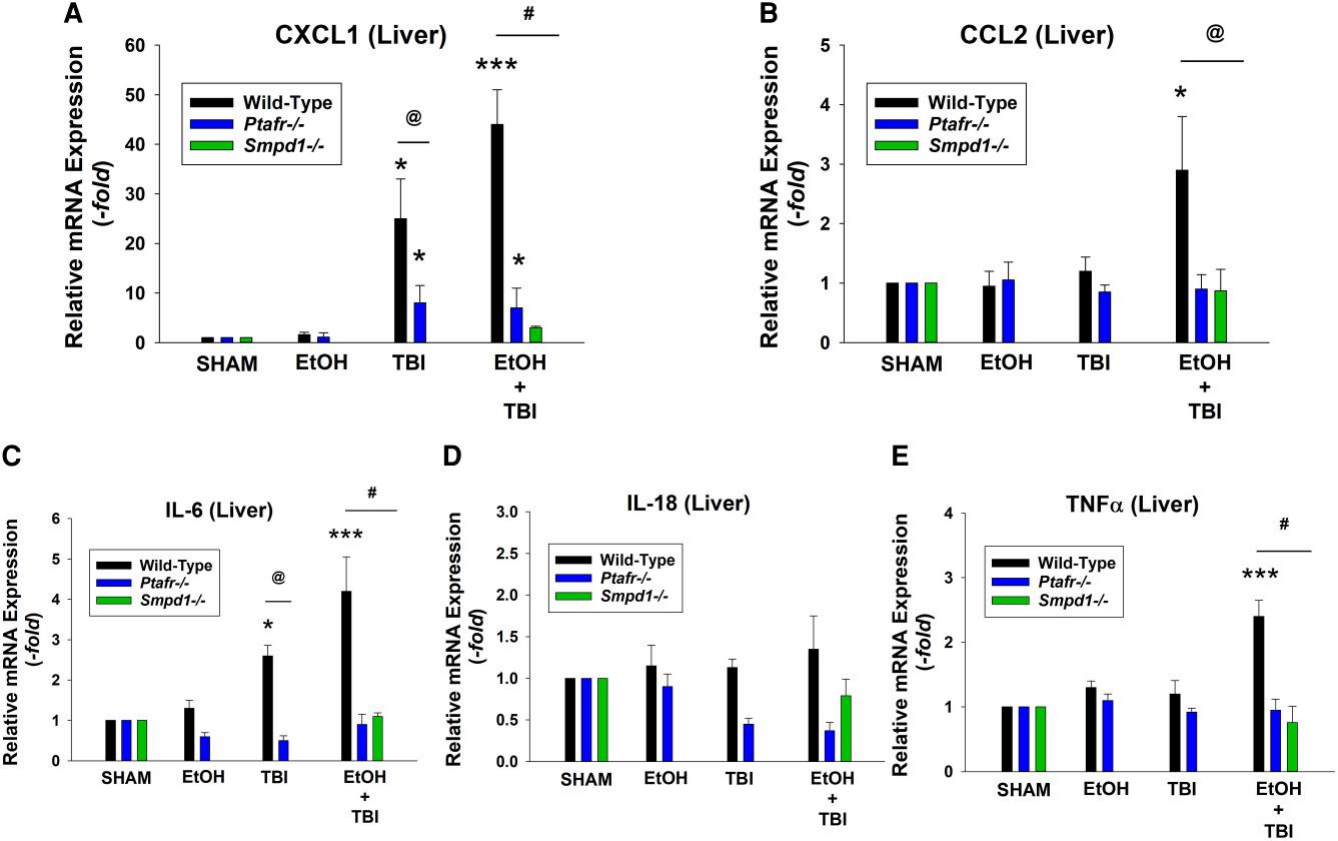
Decreased hepatic cytokine expression levels in PAFR KO and aSMase KO mice following ITBI. Mice underwent treatments as in Fig. 2, and expression levels of mRNA of representative cytokines (A: CXCL1; B: CCL2; C: IL-6; D: IL-18; E: TNF-α) in the liver were determined by quantitative real-time polymerase chain reaction, and the mean values with SEM were calculated from 10 to 15 mice in each group. The statistical analysis was performed using 2-way analysis of variance, with statistical significance denoted as *P < 0.05, **P < 0.01, and ***P < 0.001 compared with control values, with ^@^*P* < 0.05 and ^#^*P* < 0.01 indicating significant differences between similarly treated WT and KO mice.

Consistent with the histologic findings of increased leukocytes in the lung tissues, WT mice exhibited greater levels of mRNA from myeloperoxidase, as well as chemokines CXCL1 and CCL2 and cytokines IL-6, IL-18, and tumor necrosis factor α (TNF-α) in response to ITBI (Fig. 3). Of interest, thermal burn injury alone also generated increased mRNA levels of CXCL1 and IL-6 (Fig. 3B, C). As expected, given the dramatic differences in the lung histology, lungs of mice lacking PAFRs or aSMase did not contain increased levels of these cytokines in response to ITBI. Elevated levels of mRNA for cytokines CXCL1, CCL2, IL-6, and TNF-α but not IL-18 were noted in the livers of WT but not PAFR- and aSMase-deficient mice (Fig. 4). Similar findings except that IL-18 was increased along with other cytokines were noted in the kidneys and spleens (Supplementary Figs. 4 and 5). These studies suggest that the exaggerated systemic inflammatory responses in lung and other organs from the combination of ethanol and thermal burn injury reported previously^12–17^ involves the PAFR and MVP.

### 3.3 ITBI results in increased small intestinal inflammation and bacterial translocation

**Fig. 5.**
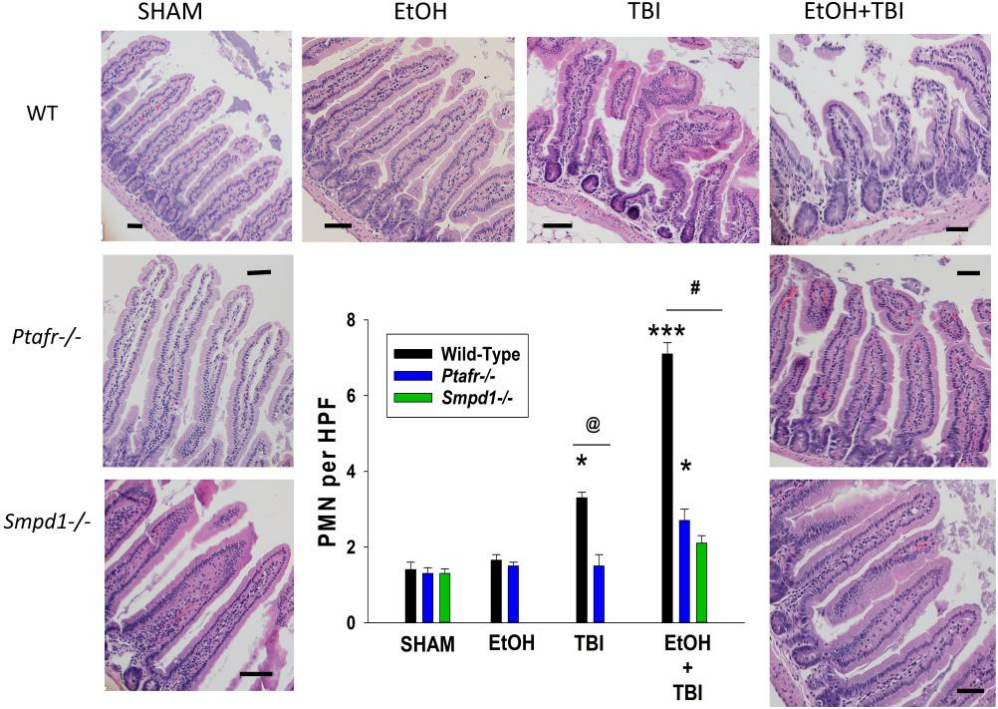
Decreased small intestinal inflammation in PAFR KO and aSMase KO mice following ITBI. One day after injury (24 h), small intestines from mice were harvested and prepared for H&E staining. The intestinal sections were examined for the degree of inflammation following ITBI in WT, PAFR KO (*Ptafr−/−*), and aSMase KO (*Smpd1−/−*) mice. Images were captured at magnification of 200× from 6 to 8 mice per group (scale bar = 20 μm). (Inset) PMN counts from 3 high-power fields (HPFs) from 6 to 8 mice taken at 600× magnitude. Statistical significance was assessed using 2-way analysis of variance. Statistically significant (*P < 0.05, ***P < 0.001) changes in MVP levels from control values (^@^*P* < 0.05, ^#^*P* < 0.01) and differences between similarly treated WT and KO mice are denoted symbolically.

**Fig. 6.**
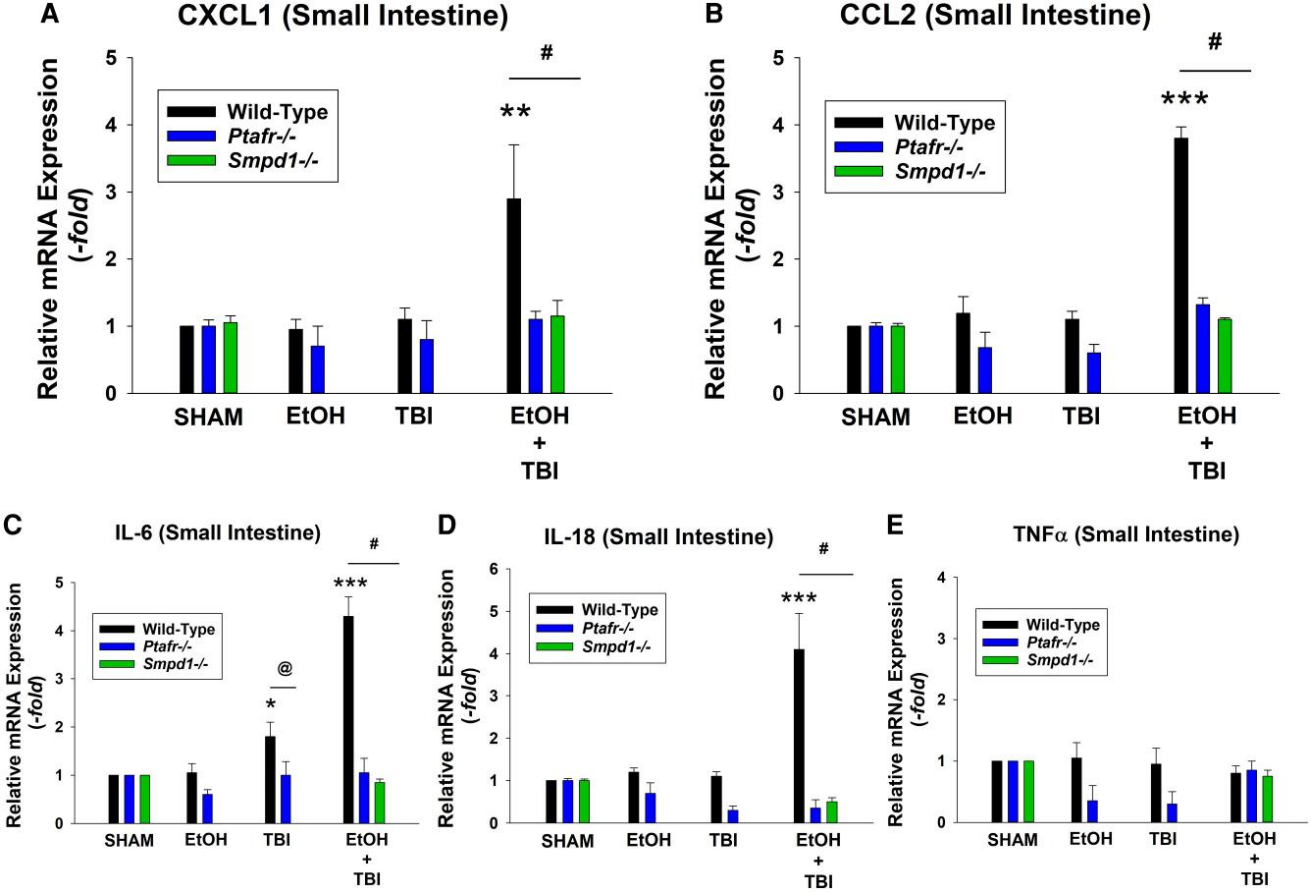
Decreased intestinal cytokine expression levels in PAFR KO and aSMase KO mice following ITBI. Mice underwent treatments as in Fig. 5, and expression levels of mRNA of representative cytokines (A: CXCL1; B: CCL2; C: IL-6; D: IL-18; E: TNF-α) in the small intestines were determined by quantitative real-time polymerase chain reaction, and the mean values with SEM were calculated from 10 to 15 mice in each group. The statistical analysis was performed using 2-way analysis of variance, with statistical significance denoted as *P < 0.05, **P < 0.01, and ***P < 0.001 compared with control values, with ^@^*P* < 0.05 and ^#^*P* < 0.01 indicating significant differences between similarly treated WT and KO mice.

**Fig. 7.**
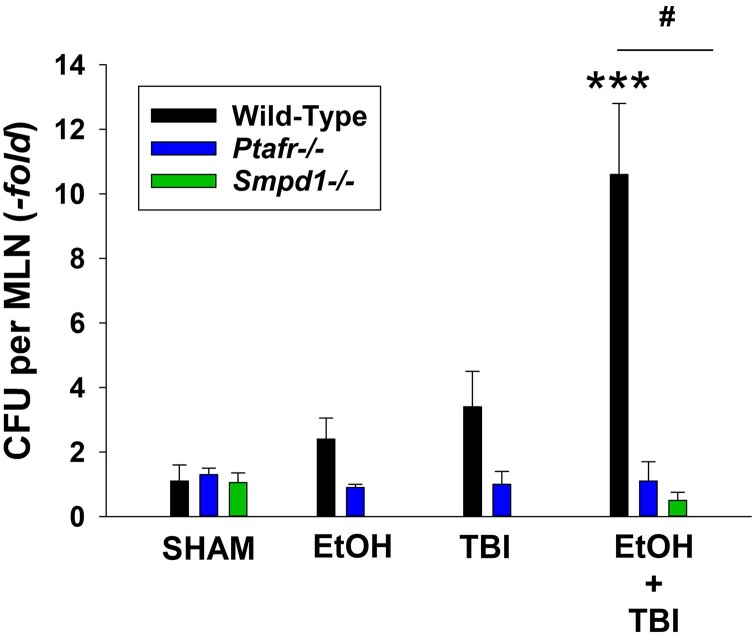
Measurement of bacterial translocation following ITBI in WT vs PAFR and aSMase KO mice. Mesenteric lymph nodes (MLNs) were isolated 1 day (24 h) after various injuries or sham treatment. The lymph nodes were homogenized and plated in triplicate on tryptic soy blood agar plates. Plates were incubated at 37 °C overnight. Colonies were counted on the next day, averaged, and divided by the lymph nodes harvested. Levels of colony-forming units (CFU)/MLN s ± SD (fold change compared with sham) from 5 to 7 mice per group. ***P < 0.001 as compared with sham or injury alone. ^#^*P* < 0.01 represents the differences between similarly treated WT and KO mice.

Previous studies have provided evidence that the multiple organ dysfunction associated with ITBI involves intestinal inflammation with resultant translocation of gut bacteria to mesenteric lymph nodes.^11,12^ This evidence includes the ability of pharmacologic inhibition of the intestinal MLCK to block both the bacterial translocation as well as the multiple organ pathology following ITBI.^11^ Moreover, toll-like receptor 4 KO mice (which would not respond to bacterial endotoxin) are protected from ITBI.^14^ Thus, the next studies tested the roles of PAFR and MVP release in the increased intestinal inflammation and bacterial translocation. As depicted in Fig. 5, 1 d post-ITBI, the histology of the small intestines from WT mice were dramatically altered with blunt villi and significantly increased numbers of neutrophils. See Supplementary Fig. 6, which depicts histology examples of 600× fields and PMNs in small intestines. The lack of PAFRs and aSMase protected the intestines from this pathologic response. The levels of mRNA of cytokines CXCL1, CCL2, IL-6, and IL-18 but not TNF-α were elevated in WT mice in response to ITBI, with attenuated responses noted in PAFR- and aSMase-deficient mice (Fig. 6). Next, the roles of PAFR and aSMase on the translocation of bacteria from gut to mesenteric lymph nodes were tested. Shown in Fig. 7, increased numbers of bacteria were found in mesenteric lymph nodes 24 h following treatment with ITBI selectively in WT mice, yet not in PAFR- or aSMase-deficient mice. An example of the primary data of bacterial plates depicting colony numbers in various genotypes in response to various treatments is found in Supplementary Fig. 7. These studies demonstrate that ITBI triggers an inflammatory response and increased bacterial translocation which is dependent upon PAFRs and the MVP-generating enzyme aSMase.

### 3.4 Ethanol + thermal burn injury triggers the formation of MVP containing high levels of PAFR agonists

**Fig. 8.**
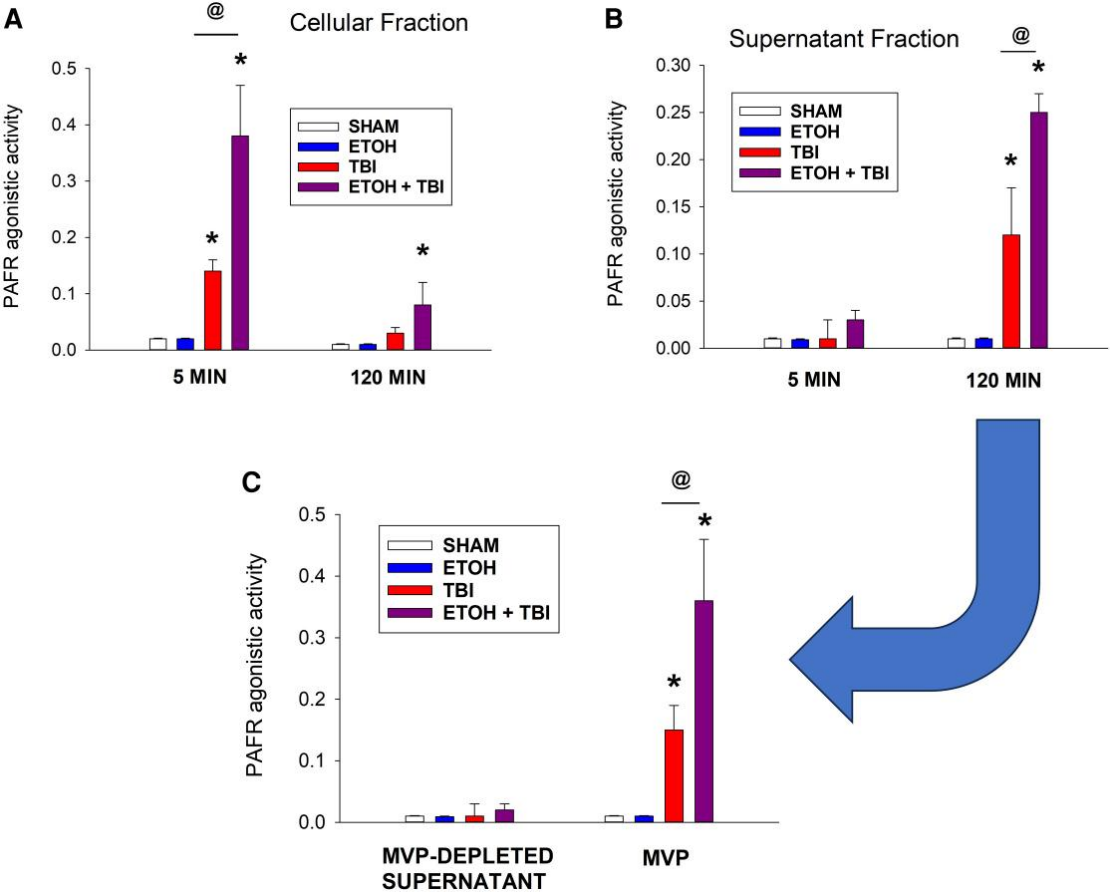
Evidence for PAFR agonistic activity in ITBI-generated MVP in HaCaT keratinocytes. HaCaT keratinocytes were subjected to no treatment (SHAM), 1% EtOH, 30-s thermal burn injury (TBI), or 1% EtOH for 30 min followed by TBI (ITBI). At 5 and 120 min, the supernatants were removed, and plates were treated with ice-cold methanol and water (1:1). The supernatants underwent centrifugation to remove cells, and these were added to the cellular fraction. Lipids were extracted and added to KBP cells for a 12-h incubation, and supernatants were removed from the KBP cells and assayed for IL-8 protein by enzyme-linked immunosorbent assay. The figures depict PAFR activity in (A) cellular fractions, (B) supernatant fractions, and (C) supernatants at 120 min posttreatment (see arrow) in which the MVP fractions isolated from the supernatants or resultant supernatants were depleted of MVP. The data are mean ± SE PAFR activity normalized to 1 nM CPAF and 5 × 10^6^ HaCaT cells from 3 separate experiments. The statistical analysis was performed using 2-way analysis of variance, with statistical significance denoted as *P < 0.05 compared with control values, with ^@^*P* < 0.05 indicating significant differences between treatment groups.

An explanation for the current findings that ITBI-induced multiple organ inflammation is dependent on PAFR and aSMase could be that the MVP shed from the skin could be carrying PAFR agonists. Of importance, our previous studies have demonstrated that MVP released by HaCaT keratinocytes in response to thermal burn injury carry PAFR agonists.^22^ To define the effect of ethanol combined with thermal burn injury on PAFR agonistic activity of MVP, we treated HaCaT keratinocytes with sham, ethanol, or thermal burn injury alone, or their combination. At 5 and 120 min posttreatment, we obtained cells and cell-free supernatants and extracted the lipids and tested them for total PAFR biochemical activity by treatment with PAFR-positive KBP and PAFR-negative KBM cells and measuring IL-8 release as compared with that induced by 1 nM CPAF in KBP cells. The advantage of this biochemical assay is that it measures total PAFR agonistic activity.^22,23,41^ As shown in Fig. 8A and B, at 5 min postinjury, increased PAFR agonistic activity was found in the cellular but not supernatant fractions. Increased levels of PAFR activity were measured in the cellular extracts from combined ethanol + thermal burn injury over thermal burn injury alone. By 120 min, the levels of PAFR activity in the cellular fraction were only minimally elevated in the ethanol + thermal burn injury combination treatment. However, at 120 min increased PAFR agonistic activity was found in the cell-free supernatants from thermal burn injury with statistically greater amounts in the combination treatment over thermal burn injury alone. We next tested if the increased PAFR activity found in the treated supernatants resided in MVP. To that end, we tested supernatants derived from HaCaT keratinocytes subjected to the previous treatments at 120 min and isolated and tested MVP, as well as MVP-depleted supernatants for PAFR activity. Of importance, in these studies the smaller sized exosomal fractions were included with the MVP-depleted supernatants to better allow testing of specifically the MVP. As shown in Fig. 8C, the PAFR activity resided with the MVP. These studies provide evidence for the hypothesis that MVP released from the keratinocyte in response to the combination of ethanol and thermal burn injury contain large amounts of PAFR agonists.

### 3.5 Effect of topical aSMase inhibitor imipramine on the multiple-organ inflammation associated with ITBI

**Fig. 9.**
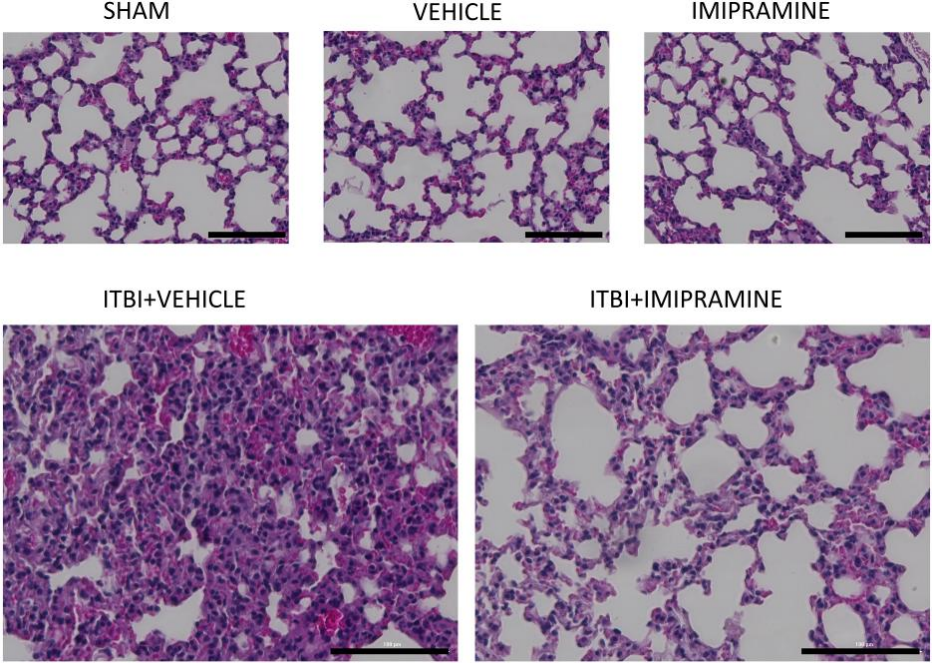
Imipramine treatment postinjury reduces alveolar wall thickening and leukocyte infiltration in lungs following ITBI. WT mice underwent treatment with sham, vehicle (90% dimethyl sulfoxide and 10% EtOH), 500 μM imipramine, ITBI + vehicle, and ITBI + imipramine. The lungs of WT mice were collected 1 d after injury and analyzed through H&E staining to investigate alveolar wall thickness and leukocyte infiltration in response to ITBI. A total of 6 to 8 mice were included in each group, and images were captured at a magnification of 200× (scale bar = 100 μm).

**Fig. 10.**
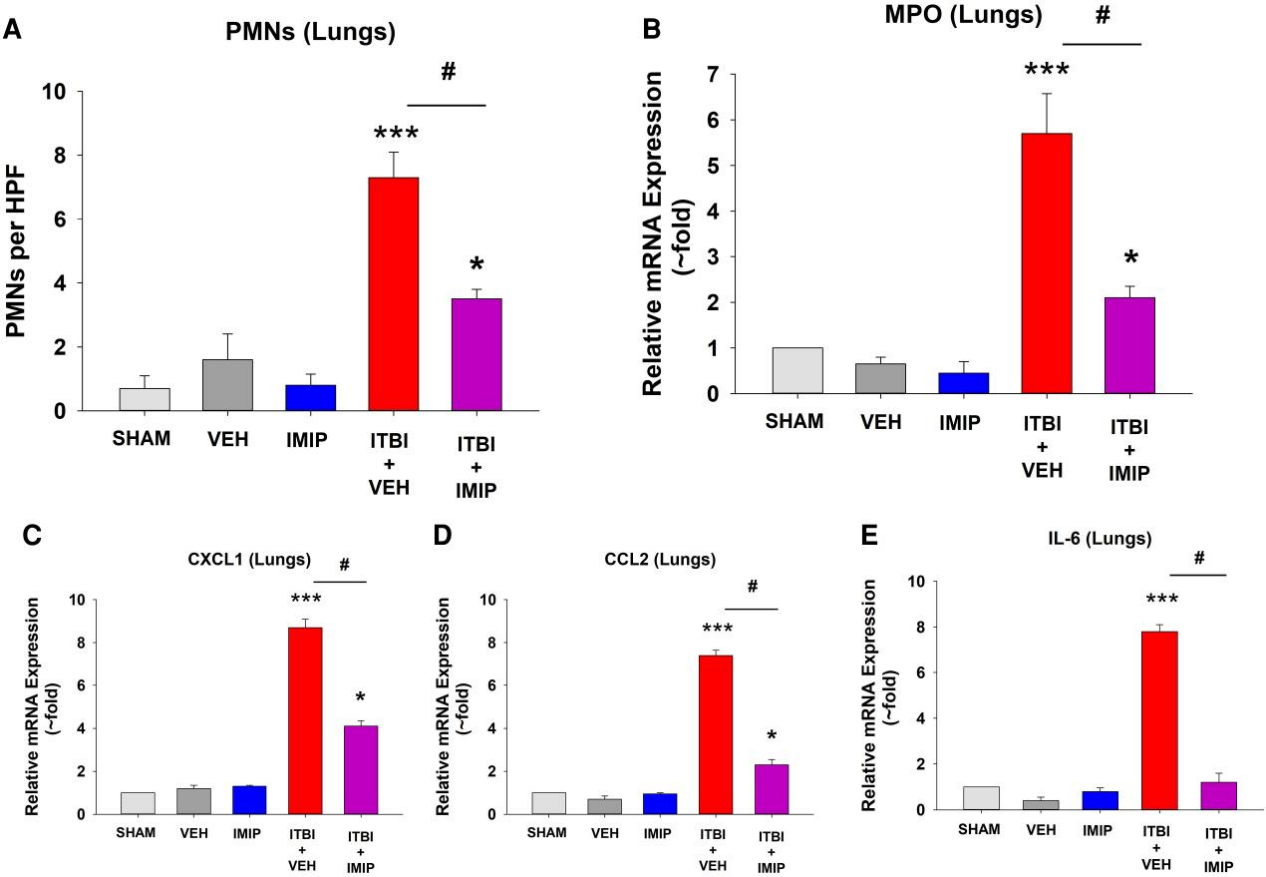
Decreased pulmonary myeloperoxidase and cytokine expression levels in response to imipramine treatment following ITBI. Mice underwent treatments as in Fig. 9, and PMN counts on 600× magnified H&E-stained tissue were obtained from 6 to 8 mice in each group (A). Expression levels of mRNA of (B) myeloperoxidase (MPO), and (C–E) representative cytokines in the lungs were determined by quantitative real-time polymerase chain reaction, and the mean values with SEM were calculated from 10 to 15 mice in each group. The statistical analysis was performed using 2-way analysis of variance, with statistical significance denoted as *P < 0.05 and ***P < 0.001 compared with sham values, with ^#^*P* < 0.01 indicating significant differences between imipramine- vs vehicle-treated WT mice subjected to ITBI. HPF = high-power field

Given our findings suggestive that the multiple-organ inflammation found in ITBI was dependent on MVP shed from skin, the next studies tested the ability of pharmacologic inhibition of aSMase to block the systemic pathologic responses. Many psychoactive drugs including tricyclic antidepressants have been demonstrated to act as potent aSMase inhibitors.^46–49^ The potential mechanism by which these weak bases exert this inhibitory effect on aSMase is that they accumulate in acidic lysosomes and detach this enzyme from the inner lysosomal membrane, where it is degraded by proteolytic enzymes.^50^ To that end, we subjected WT mice to sham vs ITBI and treated the affected skin immediately afterward with either vehicle or the aSMase inhibitor imipramine applied topically.^23,26^ The concentration of imipramine used was exactly what we have previously reported was effective in reducing MVP in mice or human skin explants in response to UVB or photodynamic therapy.^23,24^ As shown in the histologic pictures found in Fig. 9 (200×; high power), Supplementary Fig. 8 (40×; low power), and Supplementary Fig. 9 (600×; oil immersion), the lung inflammation and increased cytokine mRNA responses (Fig. 10) were attenuated by imipramine treatment. Similarly, topical imipramine also blocked the elevated levels of mRNA of inflammatory cytokines in response to ITBI in liver and small intestines (Supplementary Figs. 10 and 11, respectively). As expected, a single topical treatment with pharmacologic agent imipramine was not as effective in blocking the systemic multiple organ inflammatory response from ITBI as global KO of the aSMase enzyme. These studies not only serve to confirm the substantive role of aSMase in the ITBI pathology, but also provide a potential therapeutic to victims of this commonly encountered entity.

## 4. Discussion

Though an important source of morbidity and even mortality, the exact mechanisms by which a cutaneous thermal burn injury generates systemic effects is not clear. Not surprising, the treatment for thermal burn injuries is largely supportive in nature.^2,3^ ITBI, which has been estimated to occur in almost half of hospitalized burn injuries, appears to be a more aggressive type associated with multiple organ dysfunction.^8,12–17^ Important insights have been gleaned from murine models of ITBI, which appear to mimic the human condition.^51^ Briefly, the current picture that has emerged is that the combination of ethanol + thermal burn injury results in a systemic response characterized by MLCK activation in the intestines, which then allows bacterial translocation that then results in the widespread multiorgan inflammatory pathologies. However, many questions are as yet unanswered, to include the exact mechanism(s) by which signals from the skin reach the gut following ITBI.

**Fig. 11.**
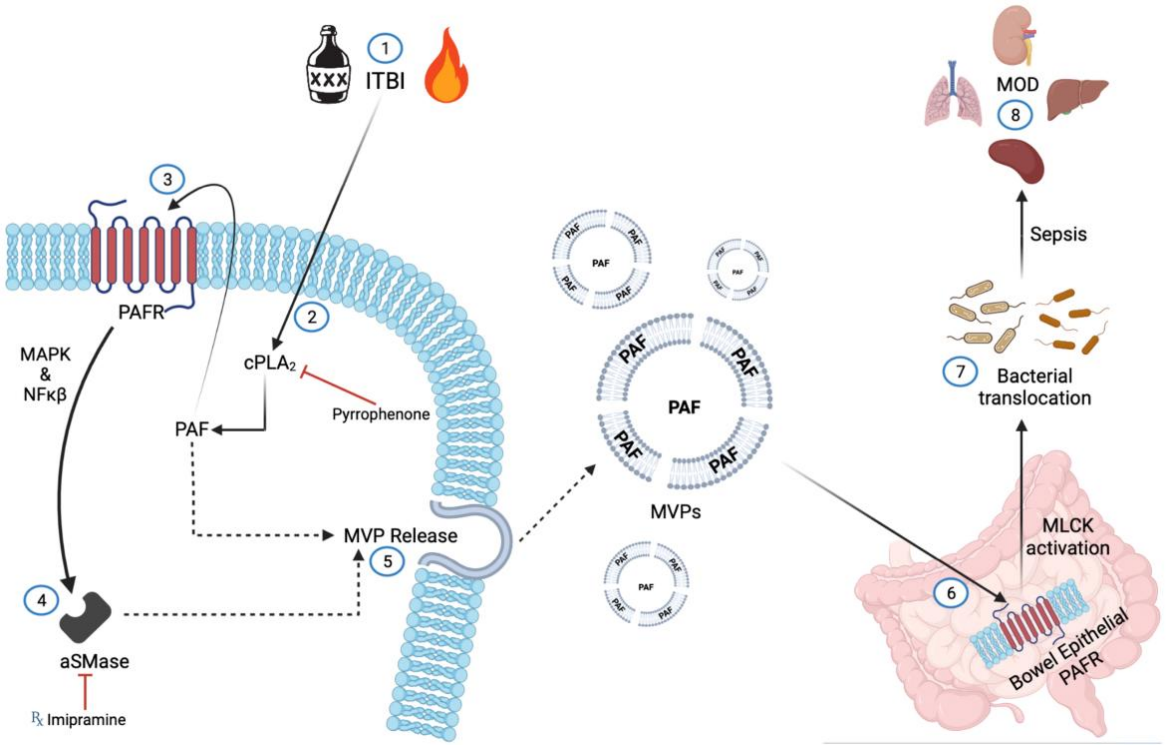
Hypothetical model by which PAF-laden MVP mediate the systemic multiple-organ inflammatory response associated with ITBI. The proposed model for the PAF system and aSMase-generated MVP in ITBI systemic effects. Ethanol and TBI combination injury (1) leads to maximized activation of cPLA_2_ (2), resulting in high levels of enzymatic PAF generation, which can trigger the PAFR within the keratinocyte cell membrane (3). Activation of the PAFR results in more enzymatic PAF production, as well as results in the translocation of aSMase via mitogen-activated protein kinases (MAPK) and nuclear factor κB (NF-κB) pathways (4). MVP generation and release following aSMase activation contain high levels of PAF in their plasma membranes, which serve to carry and protect this metabolically labile yet highly potent lipid autocoid to other regions beyond the initial site of injury (5). The MVP transports PAF to the gut, activating PAFR within the bowel epithelium (6), leading to MLCK activation, which increases gut permeability, resulting in bacterial translocation (7), and culminating in an endotoxic shock response from the seeding of organs with gut bacteria, which ultimately results in multiple organ dysfunction (MOD) from the inflammatory response (8). It should be noted that in this model ITBI could be treated pharmacologically by agents that inhibit cPLA_2_ (pyrrophenone) or aSMase (imipramine and other FIASMs) enzymes.

The current studies potentially provide a plausible mechanism involving the potent yet metabolically labile lipid mediator PAF and MVP as the agent and effector that link the skin and gut responses. Depicted in Fig. 11, we hypothesize that the increased PAF generated enzymatically by the keratinocyte in response to the combination of ethanol and thermal burn injury results in increased PAF via enhanced cPLA2 enzyme activity,^40^ resulting in the activation of the PAFR. The resulting PAFR activation not only generates more PAF in a positive, feedforward manner, but also triggers the translocation of aSMase to the plasma membrane to generate MVP.^22,23^ As PAF is found in the plasma membrane, the released MVP contain high levels of this potent lipid. The MVP likely serve to protect PAF from enzymatic degradation by both cellular and serum PAF-AHs. The PAF-laden MVP then travel systemically, to include the intestines, which then allow PAFR-mediated activation of MLCK.^52–54^ The subsequent MLCK activation results in increased intestinal permeability allowing gut flora to enter the body, which results in a sepsis-like response. The current data that support this hypothesis include the protection from ITBI-induced systemic pathology in mice lacking PAFRs or aSMase. The ability of a topical aSMase inhibitor to block the systemic effects provides support for the notion that the aSMase involved resides in the skin. The finding that the MVP from a keratinocyte cell line subjected to the combination of ethanol and thermal burn contain high levels of PAF fit with the logic that MVP is the effector.

The limitations of the current studies include that the murine models are global KOs. Tissue-specific PAFR KOs could address the exact roles of specific PAFR in keratinocytes, intestinal epithelium, and elsewhere. Our model (Fig. 11) predicts that both the keratinocyte PAFR (to generate the MVP) and the intestinal epithelial PAFR (for MLCK activation) are needed for the systemic pathology. Similarly, our model predicts, and the pharmacologic use of topical imipramine supports, that the aSMase needed for the MVP generation resides in the keratinocyte.

The current findings providing a mechanism for the increased pathologies associated with ITBI suggest that ethanol intoxication allows a more vigorous response to a burn injury via generating PAF-laden MVP. Yet, thermal burn injury alone can generate both PAF and MVP.^22,37,40^ We predict that extensive burn injuries (over the current ∼15% body surface area model) could generate this response even without the synergistic agent ethanol present.^7^ Moreover, it is entirely possible that other types of epithelial-based injuries from agents such as radiation, cold injury, or toxicants including sulfur mustargens could also result in systemic signaling using this mechanism. This previously unappreciated pathway could have relevance for both skin and other epithelial-lined surfaces such as the lung and upper respiratory tract.

The current studies also provide a potential treatment for ITBI, and possibly TBI. There are multiple functional inhibitors of aSMase (FIASMs) that have structural features that are postulated to bind to the enzyme and disrupt its trafficking.^46,47,50^ The present model of application of the topical FIASM immediately following ITBI would not be a clinically feasible option. Future studies can address the optimal FIASM and route of administration,^48,49^ as well as the time postinjury that this intervention could be effective.

## Supplementary Material

qiae078_Supplementary_Data
