# Supplementary material for: Evidence that keratinocyte microvesicle particles carrying platelet-activating factor mediate the widespread multiorgan damage associated with intoxicated thermal burn injury
Source: J Leukoc Biol. 2024 Mar 26;116(4):766–78. doi: 10.1093/jleuko/qiae078 (PMC11444260; doi:10.1093/jleuko/qiae078)
Supplement: qiae078_Supplementary_Data [file qiae078_supplementary_data.pdf]

Supplementary Figures
Lohade-Brewer et al.

Figure S1. Decreased MVP release in skin in aSMase KO mice following ITBI compared to Wild-type mice.
Figure S2. Decreased pulmonary inflammation in PAFR KO and aSMase KO mice following ITBI.
Figure S3. Attenuated ITBI-induced neutrophil infiltration in the lungs of PAFR KO and aSMase KO mice.
Figure S4. Decreased renal cytokine expression levels in PAFR KO and aSMase KO mice following ITBI.
Figure S5. Decreased splenic cytokine expression levels in PAFR KO and aSMase KO mice following ITBI.
Figure S6. Histological analysis of neutrophil infiltration in the small intestines.
Figure S7. Examples of TSA plates measuring bacterial translocation following ITBI.
Figure S8. Imipramine treatment post-injury reduces alveolar wall thickening and leukocyte infiltration in lungs following ITBI.
Figure S9. Imipramine treatment post-injury reduces PMN infiltration in lungs following ITBI.
Figure S10. Decreased hepatic cytokine expression levels in response to imipramine treatment following ITBI.
Figure S11. Decreased intestinal cytokine expression levels in response to imipramine treatment following ITBI.

Supplementary Table I List of primers used.

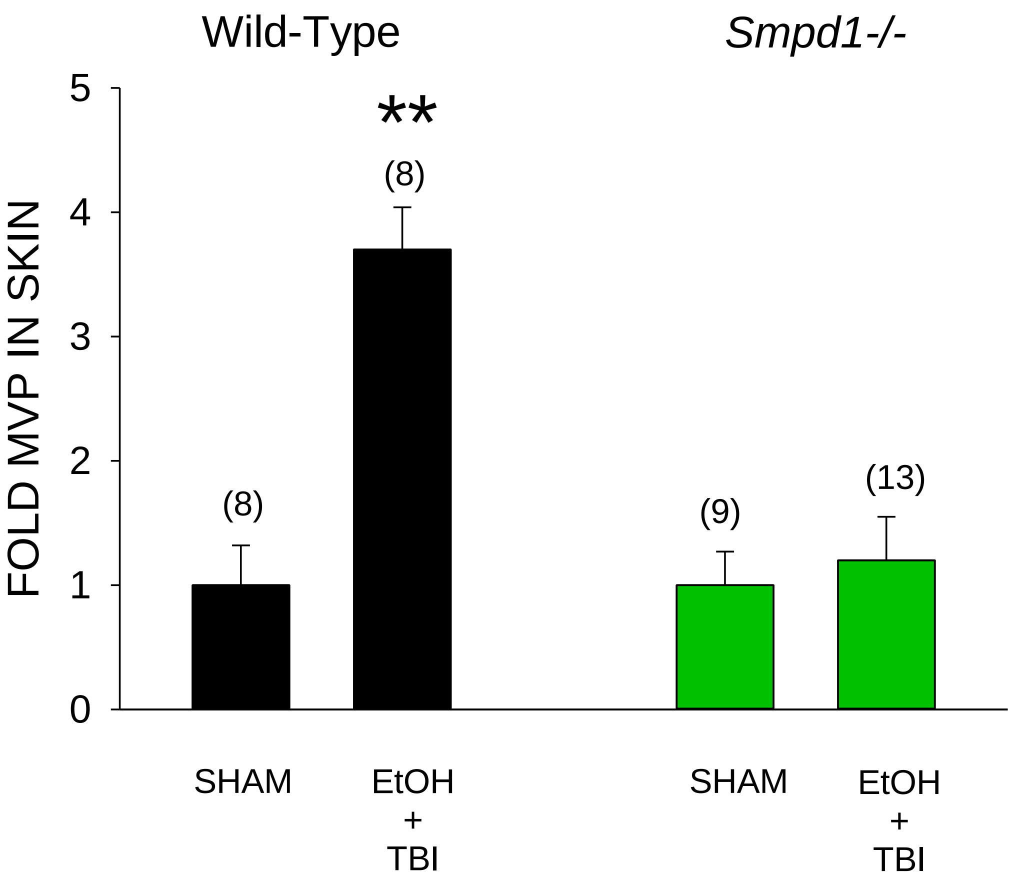

Supplementary Figure S1. Decreased MVP release in skin in aSMase KO mice following ITBI compared to Wild-type mice. Wild-type and aSMase KO (*Smpd1-/-*) mice underwent ITBI or no treatment (SHAM), and 2 hours later duplicate skin biopsies were obtained. MVP were measured as per our previous protocol (REF 23), and normalized to tissue weight (g). The mean values with SEM of fold-change were calculated from 8-13 mice in each group. Baseline levels of MVP in the Wild-type mice were 3.0 (SEM 0.3) x 10^7 MVP/g and 9.6 (SEM 0.6) x 10^7 MVP/g in the aSMase KO mice. The statistical analysis was performed using two-way ANOVA, with statistical significance denoted as $^{**}P < 0.01$ compared to control values.

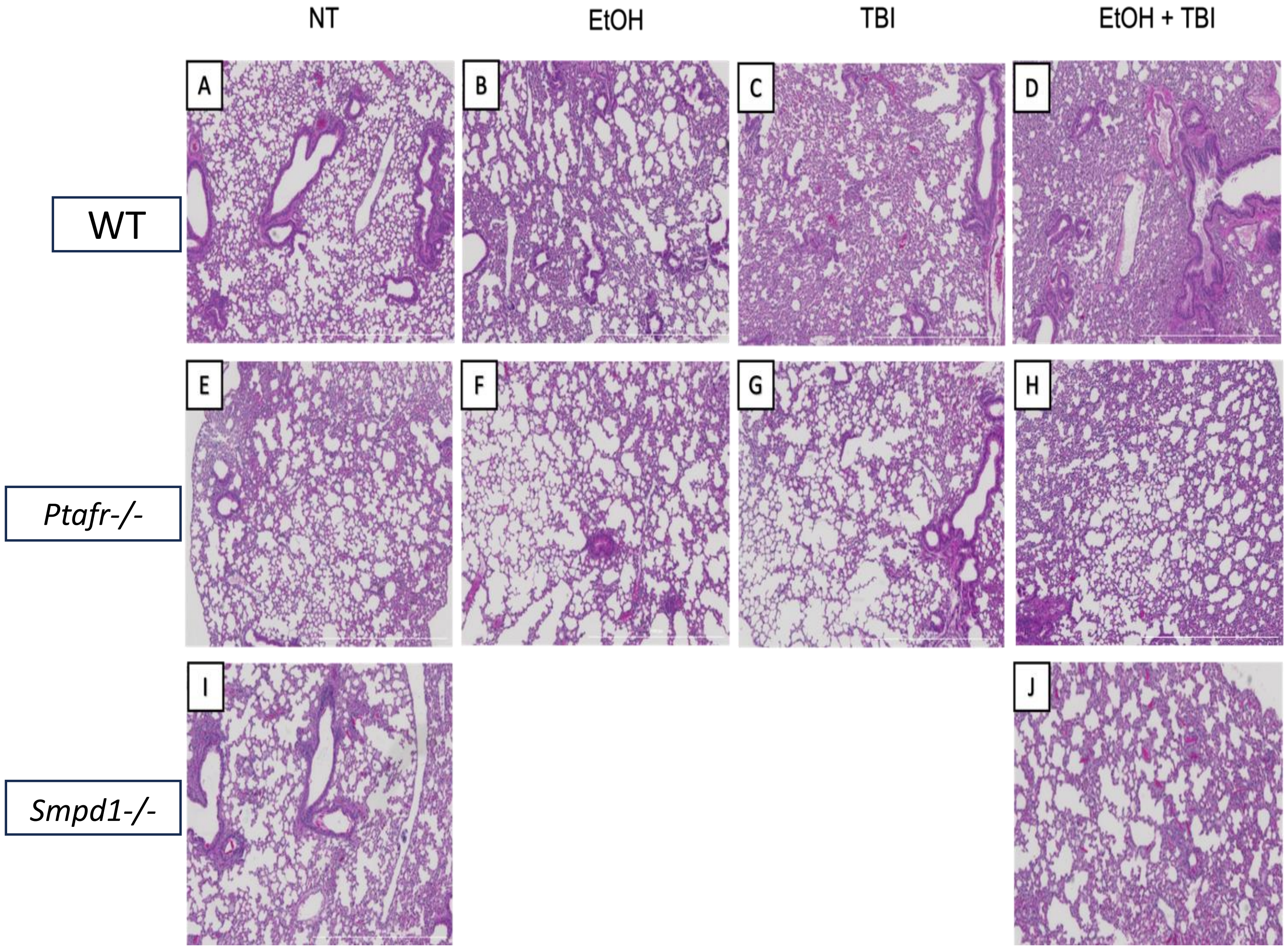

Supplementary Figure S2. Decreased pulmonary inflammation in PAFR KO and aSMase KO mice following ITBI. One day after injury (24 hours), lungs from mice were harvested and prepared for H&E staining. The lung sections were examined for the degree of inflammation following ITBI in wild-type (WT A-D), PAFR KO (*Ptafr-/-* E-H), and aSMase KO (*Smpd1-/-* I-J) mice. Images were captured at magnification of 40x from 6-8 mice per group.

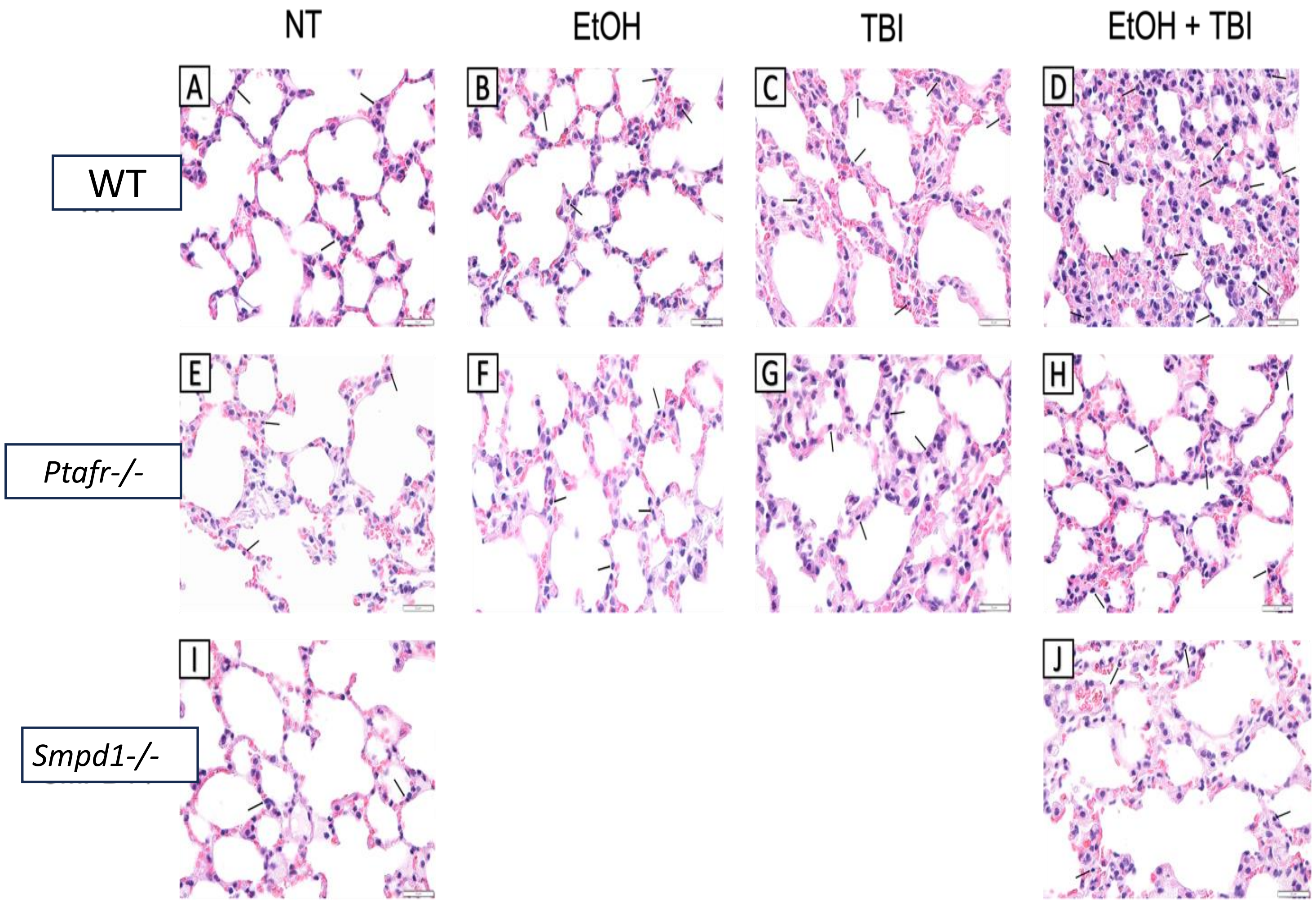

Supplementary Figure S3. Attenuated ITBI-induced neutrophil infiltration in the lungs of PAFR KO and aSMase KO mice. The images of lung sections were captured at magnification of 600x from (A-D) wild-type, (E-H) PAFR KO, and (I-J) SMPD1 KO mice. The sections were subsequently analyzed to detect the presence of neutrophils based on their morphology, and the arrows indicate the infiltrated neutrophils. N = 6-8 mice per group.

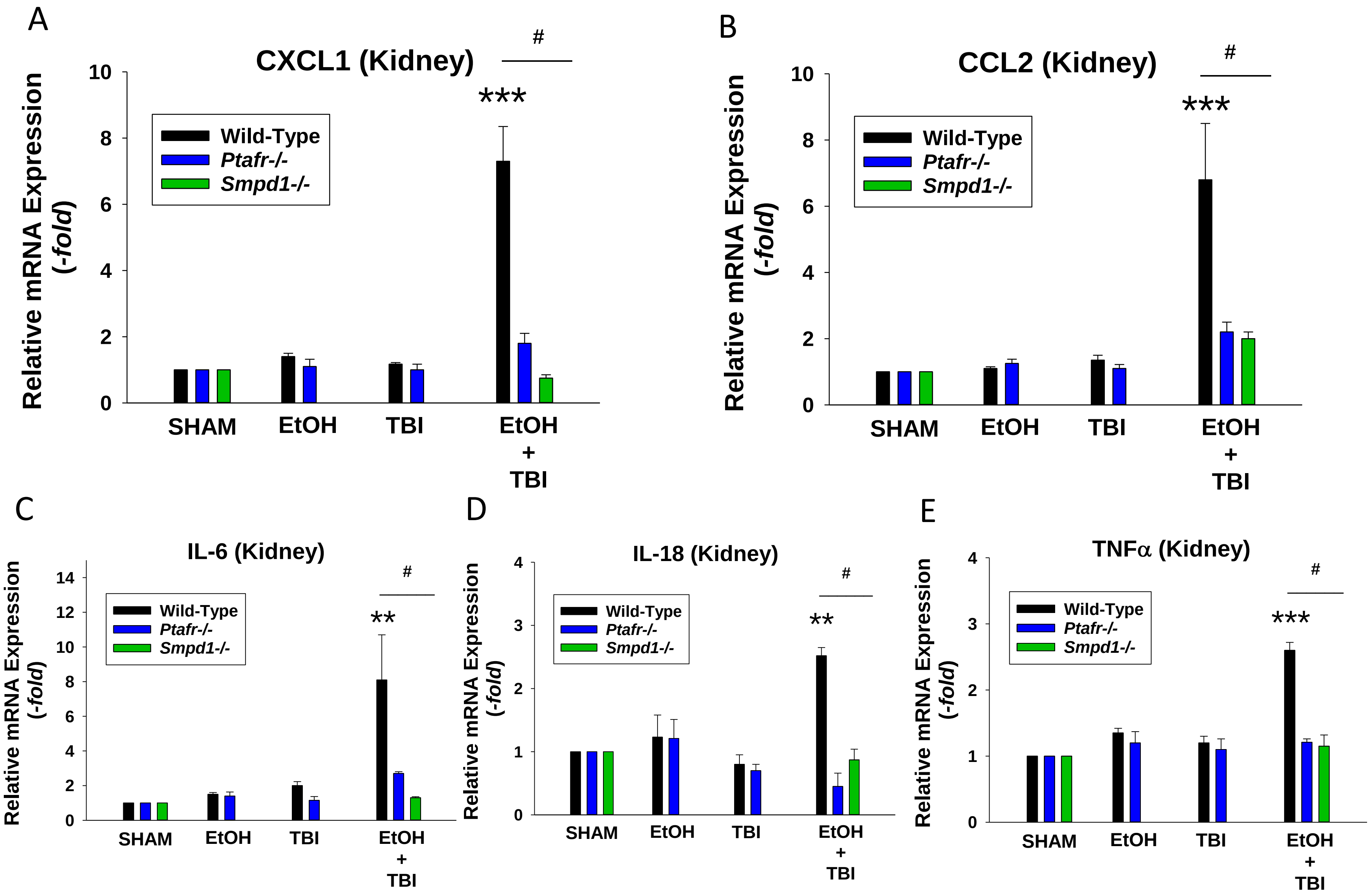

Supplementary Figure 4. Decreased renal cytokine expression levels in PAFR KO and aSMase KO mice following ITBI. Mice underwent treatments as in Figure 2, and expression levels of mRNA of representative cytokines in the kidneys were determined by RT-qPCR, and the mean values with SEM were calculated from 10-15 mice in each group. The statistical analysis was performed using two-way ANOVA, with statistical significance denoted as *p < 0.05, ***P < 0.01*, and ****P < 0.001* compared to control values, $^{\#}$*P < 0.01* indicating significant differences between similarly treated WT and KO mice.

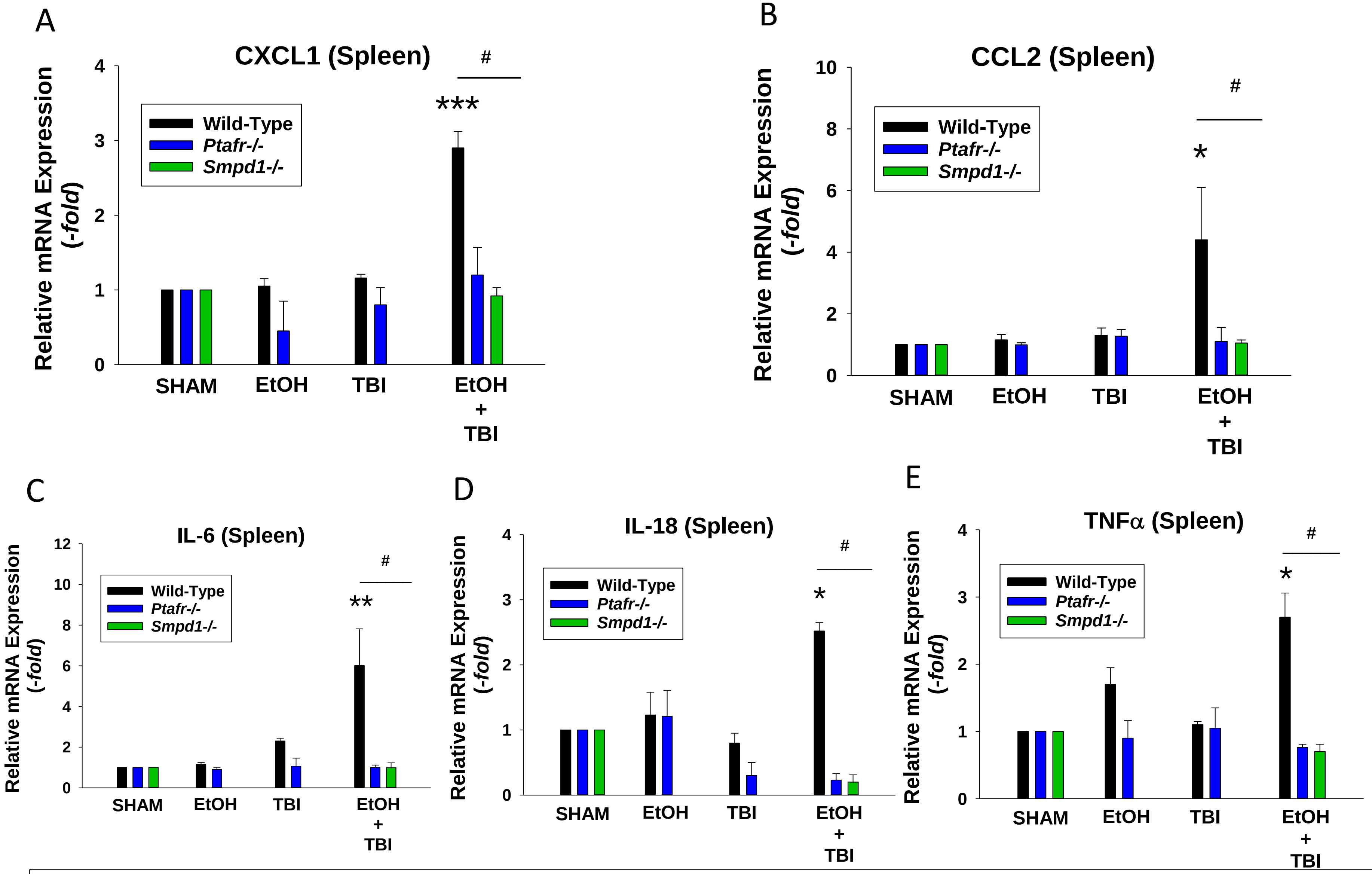

Supplementary Figure 5. Decreased splenic cytokine expression levels in PAFR KO and aSMase KO mice following ITBI. Mice underwent treatments as in Figure 2, and expression levels of mRNA of representative cytokines in the spleenss were determined by RT-qPCR, and the mean values with SEM were calculated from 10-15 mice in each group. The statistical analysis was performed using two-way ANOVA, with statistical significance denoted as *$p < 0.05$, **$P < 0.01$, and ***$P < 0.001$ compared to control values, $^{\#}P < 0.01$ indicating significant differences between similarly treated WT and KO mice.

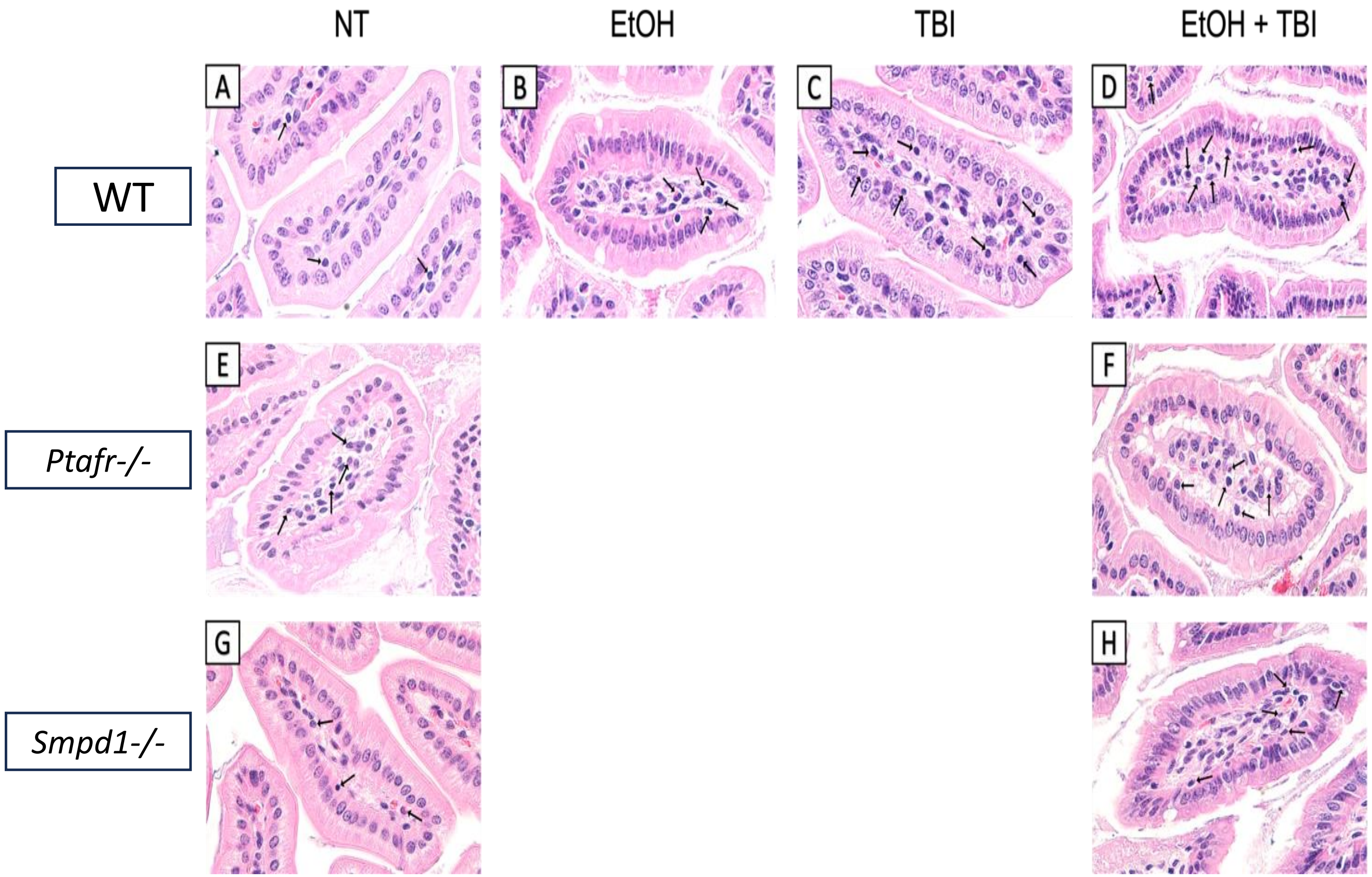

Supplementary Figure S6 Histological analysis of neutrophil infiltration in the small intestines. Intestines were collected and prepared for hematoxylin-eosin staining one day after the injury. The sections were examined under a magnification of 600x and captured as photomicrographs. Representative images of the mice intestines from different groups were analyzed for the presence of neutrophils, including (A-D) Wild-type, (E, F) PAFR KO, and (G, H) aSMase KO mice. The arrow indicates neutrophils (N=6-8 mice per group).

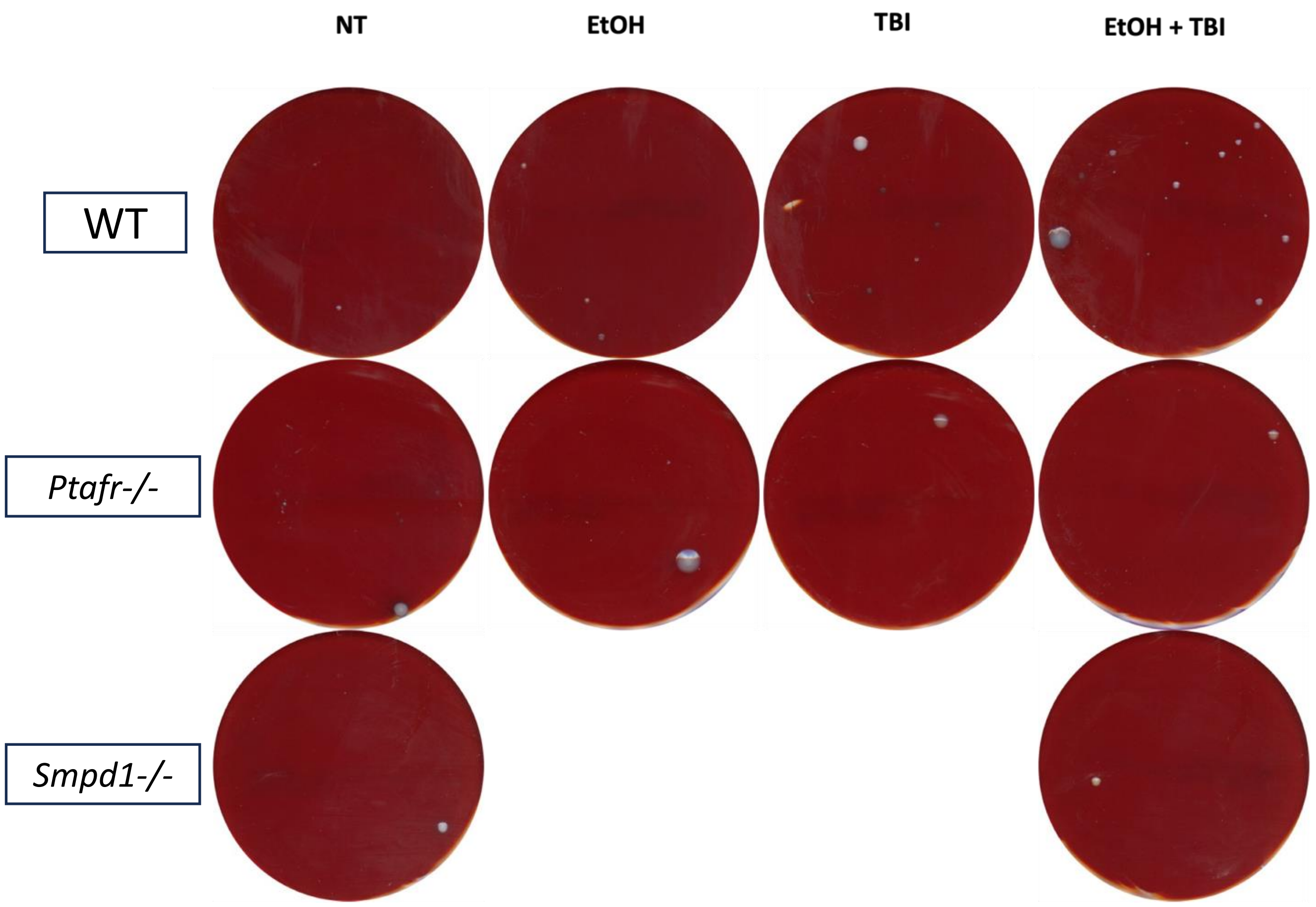

Figure Supplemental S7. Examples of TSA plates measuring bacterial translocation following ITBI. Mesenteric lymph nodes were isolated one day (24 hours) after various injuries or sham treatment. The lymph nodes were homogenized and plated in triplicate on tryptic soy blood agar (TSA) plates. Plates were incubated at 37°C overnight. Colonies were counted on the next day, averaged, and divided by the lymph nodes harvested. Blood agar plates showing bacterial colonies.

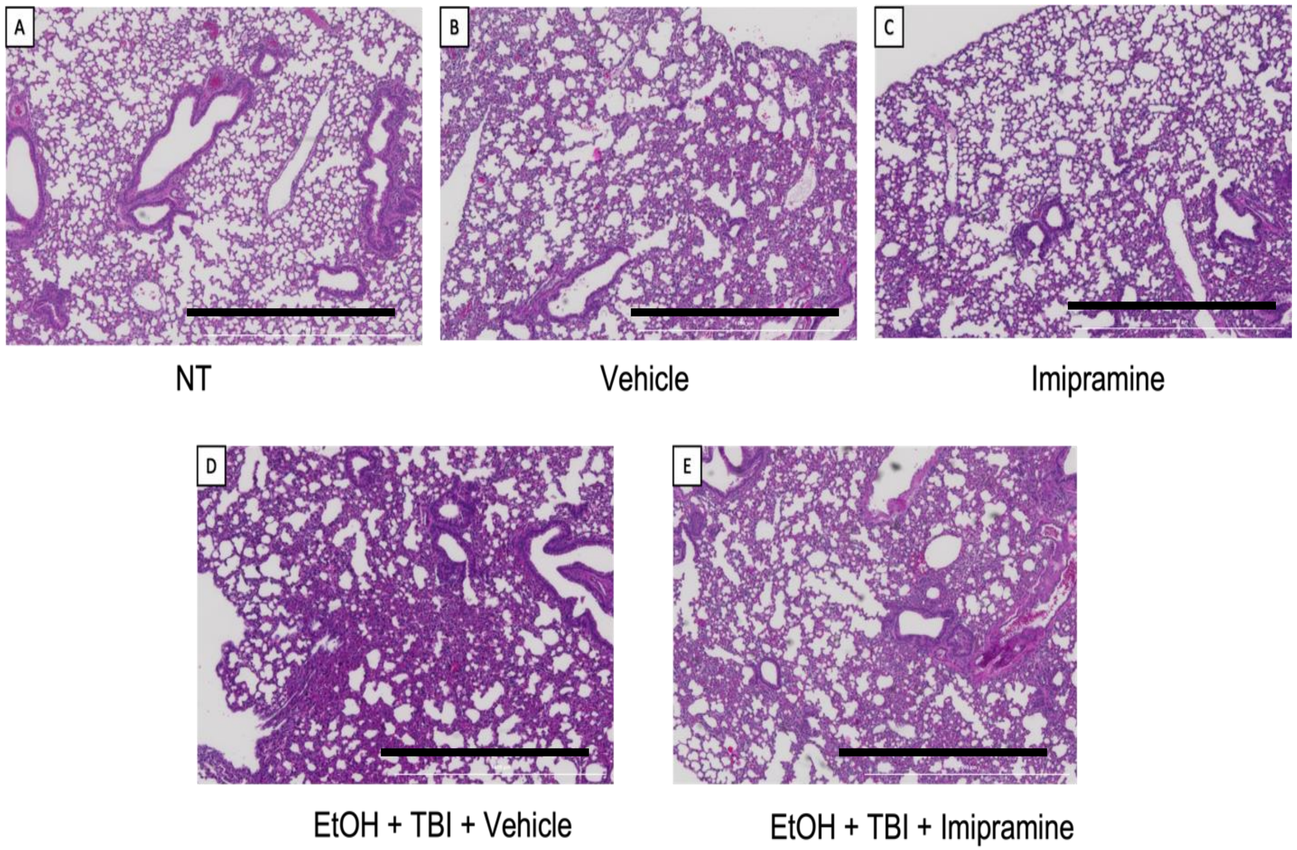

Supplementary Figure S8. Imipramine treatment post-injury reduces alveolar wall thickening and leukocyte infiltration in lungs following ITBI.

Wild-type mice underwent treatment with Sham, Vehicle (90% DMSO and 10% EtOH), 500 μM Imipramine, ITBI + Vehicle, and EtOH + TBI (ITBI) + Imipramine. The lungs of WT mice were collected one day after injury and analyzed through H&E staining to investigate alveolar wall thickness and leukocyte infiltration in response to ITBI. A total of 6-8 mice were included in each group, and images were captured at a magnification of 40x (Bar – 1000 μm).

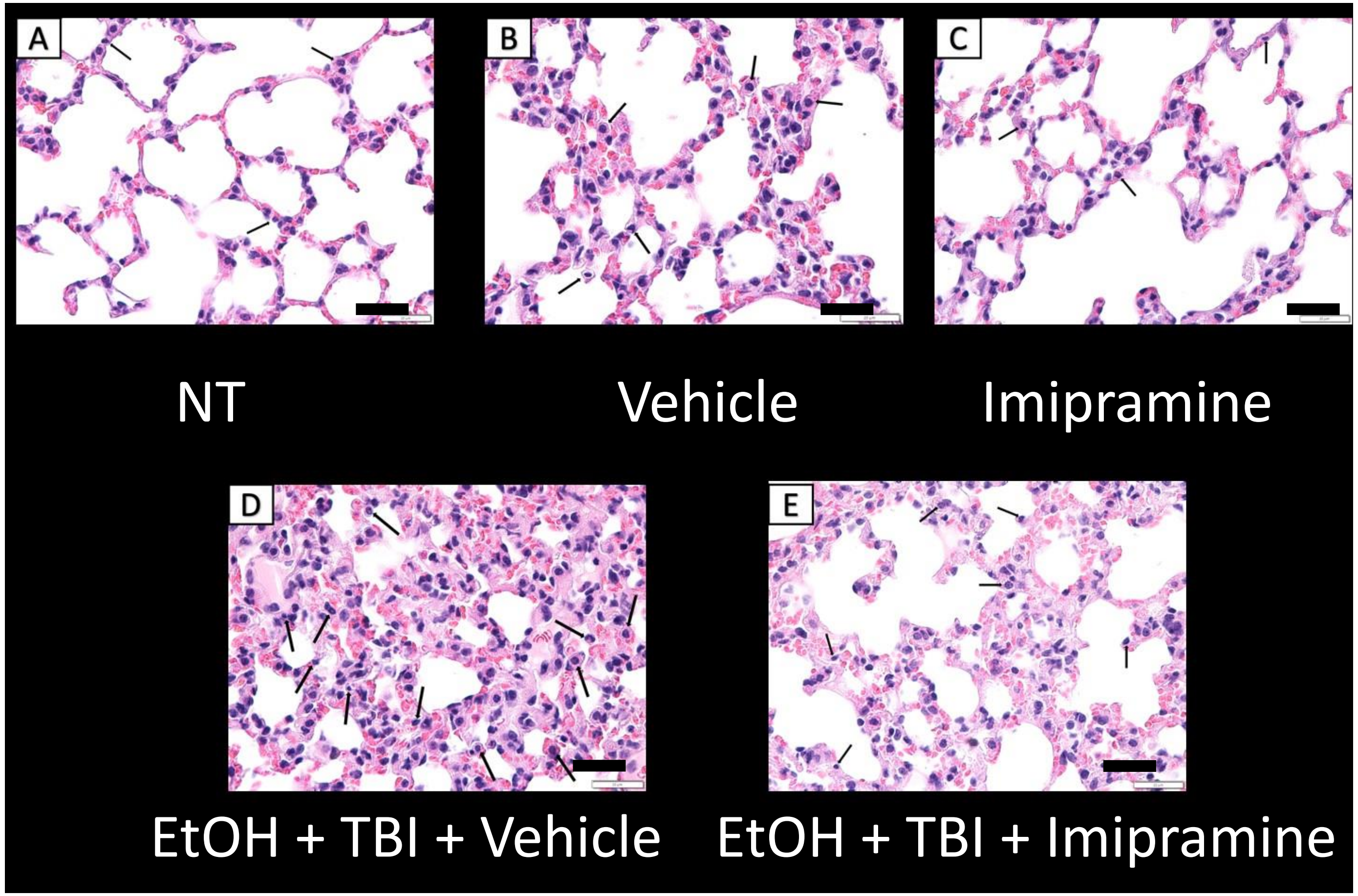

Supplementary Figure S9. Imipramine treatment post-injury reduces PMN infiltration in lungs following ITBI. Wild-type mice underwent treatment with (A) Sham, (B) Vehicle (90% DMSO and 10% ETOH), (C) 500 μM Imipramine, (D) EtOH + TBI (ITBI) + Vehicle, and (E) EtOH + TBI (ITBI) + Imipramine. The lungs of WT mice were collected one day after injury and analyzed through H&E staining at 600x to investigate the presence of neutrophils based on their morphology, and the arrows indicate the neutrophils. N = 6-8 mice per group. (Bar- 20 μm).

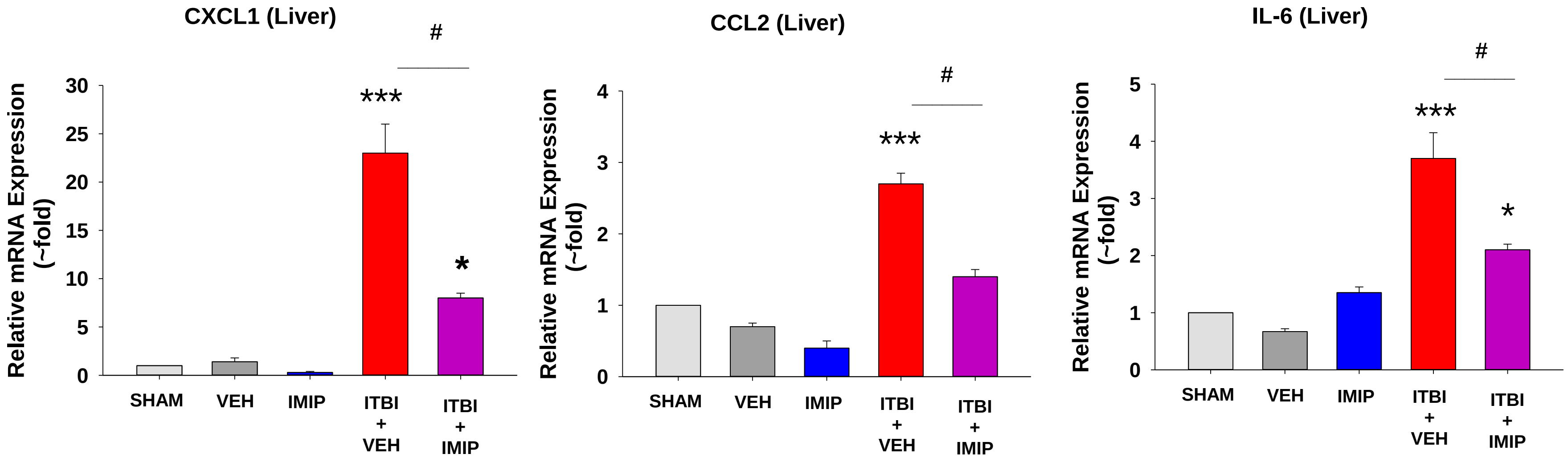

Supplementary Figure S10. Decreased hepatic cytokine expression levels in response to imipramine treatment following ITBI. Mice underwent treatments as in Figure 9. 24 h later, livers were harvested and expression levels of mRNA of representative cytokines in the lungs were determined by RT-qPCR, and the mean values with SEM were calculated from 10-15 mice in each group. The statistical analysis was performed using two-way ANOVA, with statistical significance denoted as *$p < 0.05$, and ***$P < 0.001$ compared to sham values, $^{\#}P < 0.01$ indicating significant differences between imipramine- versus vehicle-treated WT mice subjected to ITBI.

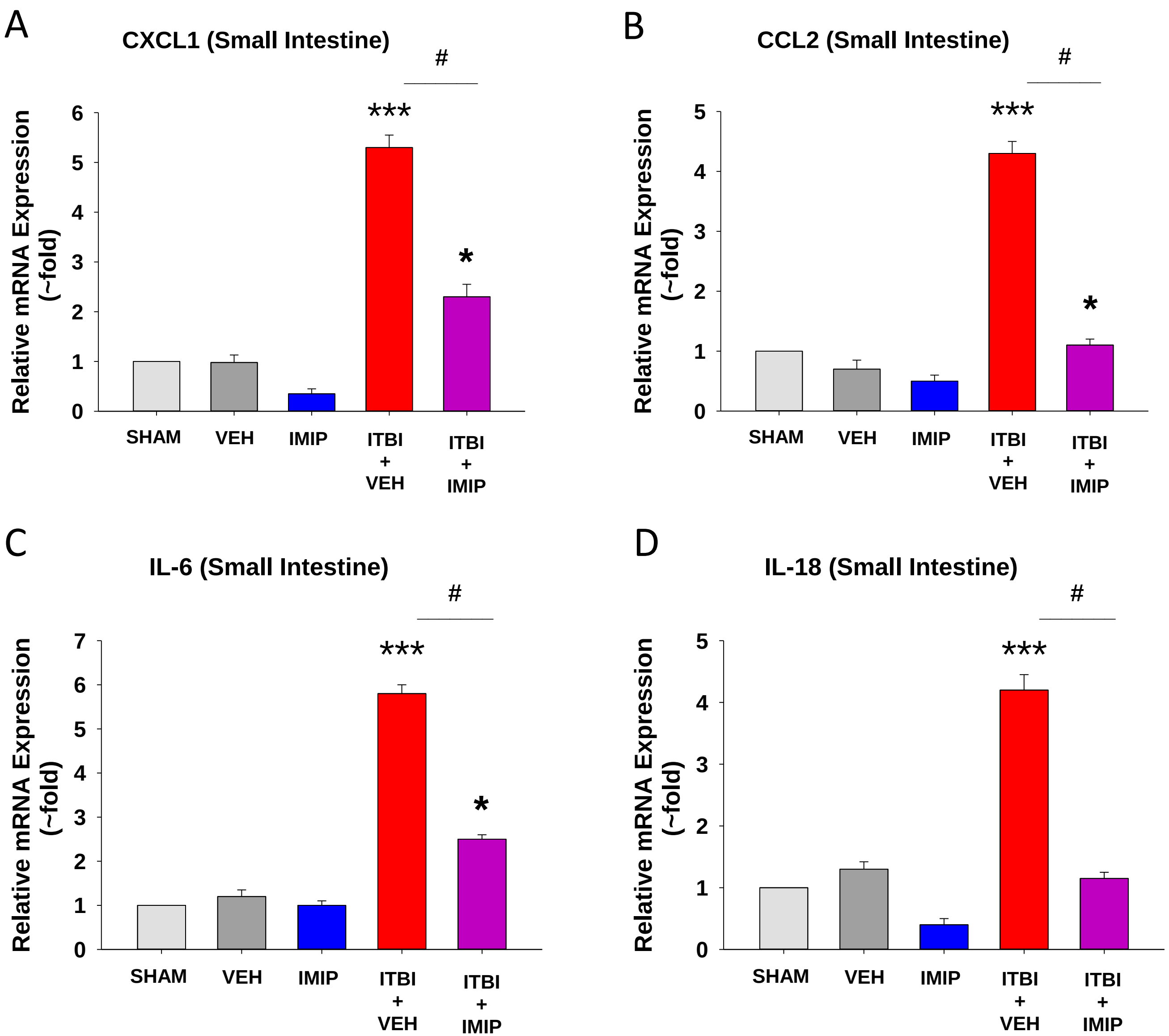

Supplementary Figure S11. Decreased intestinal cytokine expression levels in response to imipramine treatment following ITBI. Mice underwent treatments as in Figure 9. 24 h later, small intestines were harvested and expression levels of mRNA of representative cytokines in the lungs were determined by RT-qPCR, and the mean values with SEM were calculated from 10-15 mice in each group. The statistical analysis was performed using two-way ANOVA, with statistical significance denoted as *p < 0.05, and ****P < 0.001* compared to sham values, $^{\#}$*P < 0.01* indicating significant differences between imipramine- versus vehicle-treated WT mice subjected to ITBI.

Gene	Forward primer	Reverse primer
**CXCL1**	5’- TCC AGA GCT TGA AGG TGT TGC C -3’	5’- AAC CAA GGG AGC TTC AGG GTC A -3’
**IL-6**	5’- TAC CAC TTC ACA AGT CGG AGG C -3’	5’- CTG CAA GTG CAT CAT CGT TGT TC -3’
**CCL2**	5’- GCT ACA AGA GGA TCA CCA GCA G -3’	5’- GTC TGG ACC CAT TCC TTC TTG G -3’
**IL-18**	5’- GAC AGC CTG TGT TCG AGG ATA TG -3’	5’- TGT TCT TAC AGG AGA GGG TAG AC-3’
**TNF-**	5’- GGT GCC TAT GTC TCA GCC TCT T -3’	5’- GCC ATA GAA CTG ATG AGA GGG AG-3’
**MPO**	5’- CGT GTC AAG TGG CTG TGC CTA T-3’	5’- ACC GTG CCT TTG TAC GCT GGT T-3’
**Actin**	5’- TGG AAT CCT GTG GCA TCC ATG AAA C-3’	5’- TAA AAC GCA GCT CAG TAA CAG TCC G -3’

Table Supplementary I. List of primers used (Integrated DNA Technologies).
